# Improved measures for evolutionary conservation that exploit taxonomy distances

**DOI:** 10.1038/s41467-019-09583-2

**Published:** 2019-04-05

**Authors:** Nawar Malhis, Steven J. M. Jones, Jörg Gsponer

**Affiliations:** 1 Michael Smith Laboratories, University of British Columbia, Vancouver, BC Canada V6T 1Z4; 2 Michael Smith Genome Sciences Centre, BC Cancer, Vancouver, BC Canada V5Z 4S6; 3 Department of Medical Genetics, University of British Columbia, Vancouver, BC Canada V6T 1Z3; 4 Department of Biochemistry and Molecular Biology, University of British Columbia, Vancouver, BC Canada V6T 1Z3

## Abstract

Selective pressures on protein-coding regions that provide fitness advantages can lead to the regions' fixation and conservation in genome duplications and speciation events. Consequently, conservation analyses relying on sequence similarities are exploited by a myriad of applications across all biosciences to identify functionally important protein regions. While very potent, existing conservation measures based on multiple sequence alignments are so pervasive that improvements to solutions of many problems have become incremental. We introduce a new framework for evolutionary conservation with measures that exploit taxonomy distances across species. Results show that our taxonomy-based framework comfortably outperforms existing conservation measures in identifying deleterious variants observed in the human population, including variants located in non-abundant sequence domains such as intrinsically disordered regions. The predictive power of our approach emphasizes that the phenotypic effects of sequence variants can be taxonomy-level specific and thus, conservation needs to be interpreted accordingly.

## Abstract (web summary)

Information on protein sequence variability and conservation can be leveraged to identify functionally important regions. Here, the authors develop new conservation measures that exploit taxonomy distances and LIST, a tool for predicting deleteriousness of human variants.

## Introduction

Directional selection leads to an increase in the frequency of alleles that provide fitness advantages and their conservation through speciation events and genome duplications^1^. Knowledge of this evolutionary conservation is exploited across all the biosciences to characterize the structure^2–4^, function^5–7^, interactions^8–11^, and regulation^12–15^ of proteins. Furthermore, conservation information has been used to redesign proteins^16,17^ as well as to establish their evolutionary trajectories and relationships^18,19^. Different measures have been developed to calculate evolutionary conservation scores from multiple sequence alignments (MSAs), the most popular of which exploit variant frequencies in the MSAs^20^ and/or phylogenetic relationships among preselected subsets of species^21^. Computational methods successfully exploit these measures to quantify conservation of protein positions and predict the deleteriousness of variants. Some methods rely solely on conservation measures to predict the deleteriousness of variants (e.g., SIFT^22^, PROVEAN^23^, EVmutation^24^, phyloP^25^, and GERP++^26^), whereas others complement conservation measures with features derived from functional genomic and gene annotation data or are supplemented by orthogonal prediction methods (e.g., PolyPhen-2^27^, CADD^28^, Eigen^29^, DANN^30^, and fitCons^31^). Despite the success of these tools, an overreliance on similarly flavored conservation measures will only permit slight, incremental progress in the prediction of deleteriousness of protein-coding variants, highlighting the need for new conservation measures.

We sought to develop new conservation measures based on the following concepts and ideas. The contribution of a protein to the observed phenotype is a complex function that depends on proper folding and activity as well as cellular localization and interactions with partners. Importantly, this function is specific to the cellular environment of each species, and differences in the environment of homologous proteins are likely to increase with the taxonomic distance between the species. Thus, when assessing the deleteriousness of a human amino-acid variant, it is important to not only evaluate whether a matching variant has already been observed in homologs, but also how closely related the species with the matching variant are, which often correlates with the similarity between the human and these species’ genomes. We hypothesize that a variant to a human gene that already exists in the reference sequence of another species is more likely to be benign when that species is closely related to human, whereas the variant is more likely to be deleterious when it is observed in a distant species. While the first part of the hypothesis is intuitive, support for the second part comes from the following observations. Systematic analyses examining the conservation of phosphosite residues in proteins have revealed that some of these residues  are highly conserved in higher eukaryotes but replaced by phospho-mimicking aspartic or glutamic acid in homologous proteins in lower eukaryotes, prokaryotes and archea^32^. Importantly, various phospho-mimicking gain-of-function variants are known to trigger constitutive activation of proteins and drive cancerous cell transformation in humans^33^. Thus, amino acids that are present in the protein reference sequence of species that are far from human in the taxonomy tree may cause disease when present in a human. Consequently, we propose that measures exploiting the closeness of species are more effective in the assessment of conservation of sequence positions, and thus the deleteriousness of variants, than classical conservation measures, specifically those that rely on variant frequencies across species.

Here, we introduce novel conservation measures. These measures are used to create LIST, a method that predicts deleteriousness of human variants in protein-coding regions based on Local Identity and Shared Taxa. LIST predictions show a substantial improvement over methods that rely solely on previously established conservation measures while also outperforming methods that combine conservation measures with gene annotations and genomic features.

## Results

### Taxonomy-based conservation measures

**Fig. 1:**
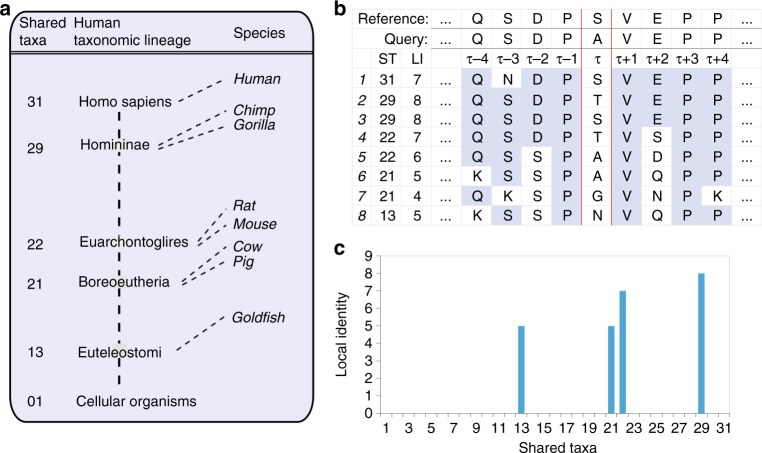
New conservation measures based on alignment identity and taxonomy distances. **a** Simplified taxonomy tree. Shared taxa (ST) is defined as the number of taxonomy tree edges that are shared between human and another species. Goldfish, for instance, shares 13 edges with the human taxonomic lineage, and thus its ST value is 13. It is important to note that a given taxa can include multiple species. For instance, shared taxa 22 contains mouse, rat and other rodents not listed as well as lagomorphs, treeshrews, colugos, and primates. The entire human taxonomy lineage can be found in Supplementary Table 1. **b** Simplified MSA used to illustrate the calculation of different LIST measures that include local identity (LI) and ST. LI for a sequence at a location *τ* is computed by counting the number of residues that are identical to the query sequence (shaded in blue) in a window size nine centered at *τ*, excluding the residue at *τ*. **c** The STP at position τ associated with the simplified MSA presented in **b**

**Fig. 2:**
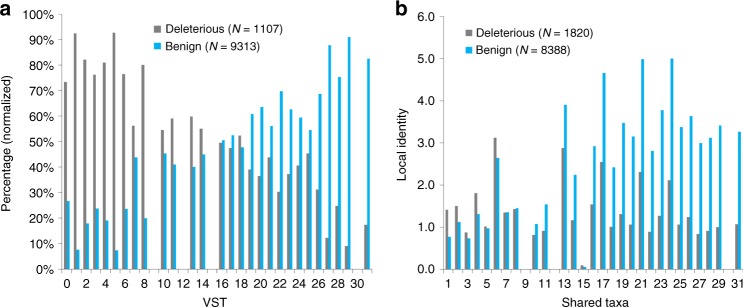
New conservation measures separate benign and deleterious variants. **a** Distribution of variant shared taxa (VST) for deleterious and benign human variants that have a matching allele in the raw MSA. For each of the 32 possible VST values that were found in the MSA analysis, the percentages of benign and deleterious variants are shown. VST values can only be calculated when a matching amino acid is found in the MSA, which defines the number of benign and deleterious variants that could be used for this plot (see methods for details on data). **b** The average shared taxa profiles (STP) of deleterious and benign variants (see methods for details on data)

An ideal data set to test our hypothesis consists of human variants that have been identified in 60,706 individuals (ExAC data)^34^ using high-throughput means (see Methods). Some of the identified variants are annotated by ClinVar as pathogenic, i.e., deleterious, while remaining variants that are observed in the human population with high frequency (≥ 1%) can be assumed benign, i.e., evolutionary neutral and not deleterious. In accordance with the ideas outlined in the introduction, the likelihood of finding a matching amino acid in homologs of species closely related to human should be lower for deleterious than benign variants. To test this corollary, we define variant shared taxa (VST) as the first measure of evolutionary conservation within our postulated framework. To calculate VST, we identify the sequence from the MSA with the amino acid matching the human variant of interest and the highest local sequence identity (LI) with the human query protein sequence (see Methods for details). We then select the sequence’s shared taxa (ST), which we define as the number of branches in the taxonomy tree that humans share with the species the sequence originates from (Fig. 1a and Supplementary Table 1). As an example, given the simplified MSA in Fig. 1b and focusing on the substitution of the reference residue S at position *τ* by amino acid A, the VST_τ,A_ is 22 because A is found in sequences 5 and 6, but sequence 5 has the highest LI with the query. We calculated VST values as well as raw frequencies in MSAs for variants with deleterious and benign effects in humans and generated histograms of the distribution of these values (Fig. 2a and Supplementary Fig. 1). These histograms support our hypothesis, namely, that variants of a human protein that exist in the reference genome of other species are more likely to be benign when these species are closely related to human but also more likely deleterious when the species are far away in the taxonomy tree. Furthermore, a comparison of VST values and raw frequencies in MSAs reveals that both measures segregate benign and deleterious variants, but that VST has a slightly higher contrast for the two classes (*r*: − 0.282 and − 0.271, Spearman rank correlation).

We then developed a second measure that assesses the variability of a sequence position across the taxonomy tree. This measure was inspired by previously developed conservation measures that derive positional entropies from the amino acid frequencies at a given sequence position. However, similar to VST, we use LI and ST in this new measure that we call shared taxa profile (STP). STP at position *τ* is a vector of *n* = 31, where each element holds the highest LI of sequences with identical ST, excluding those with amino acids matching the human reference. For the case of the simplified MSA provided in Fig. 1b, STP element 21 gets the value 5 assigned because 5 is the highest LI of all sequences with ST equal 21. Following the same rationale, element 22 gets the value 7 assigned, and so on for all other ST represented in the MSA (Fig. 1c). When averaged over all sequence positions that harbor deleterious and benign human variants, respectively, STPs (Fig. 2b) reveal a strong contrast between deleterious and benign variants. When interpreting this graph, one needs to keep in mind that STP is calculated only for positions that display sequence variations compared to the human reference. It is widely accepted that variants at non-conserved sequence positions are more likely to be benign than deleterious. Figure 2b reveals that this is more likely to be true when the sequence variations at a position have been observed in closely related species. Thus, similar to VST, STP is a powerful measure to separate deleterious and benign variants.

### Implementation of the new conservation measures in LIST

Next, we developed a new tool, LIST, which allows for a robust comparison of the predictive power of our taxonomy-based and classical conservation measures. LIST predicts the deleteriousness of human variants using three prediction modules, two of which rely on the new conservation measures VST and STP. The first module uses VST exclusively and, in essence, assesses the deleteriousness of a specific human variant by determining whether a matching amino acid occurs in a homolog of a closely or distantly-related species. The second module utilizes both VST and STP to assess how vulnerable a sequence position is to variations (see Methods for details). Finally, these two modules are complemented by a third module that exploits how likely different types of amino-acid substitutions have deleterious effects, i.e., amino-acid swap-ability. Optimal parameters for the three modules were learned from a first optimization set (Supplementary Table 2). Then, these modules were rescaled to accommodate for alignment depth and combined hierarchically^35,36^ (Supplementary Fig. 2). Rescaling and hierarchical structure parameters were learned from a second optimization set (See Methods for details). We assembled these optimization sets by using annotations and variants from ClinVar and ExAC, respectively (see Methods for details on their assembly). We contrasted LIST’s performance with that of existing methods using four different test sets that were derived from different sources (ClinVar/ExAC, UniProt/gnomAD (http://gnomad.broadinstitute.org/), Cancer (http://gnomad.broadinstitute.org/), and HumVar^27^; see Methods for details). It is important to note that variants used for optimization and testing map to different proteins, thus there is no overlap between any of the variants used in optimization and testing.

### LIST outperforms methods using existing conservation measures

**Fig. 3:**
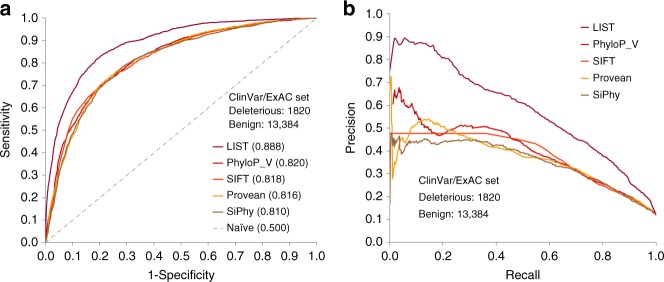
LIST performs better than other predictors in separating benign and pathogenic variants. **a** ROC curves calculated for the predictions by LIST, phyloP_Vertebrata (phyloP_V), SIFT, PROVEAN, and SiPhy on the variants from the ClinVar/ExAC test set that are scored by all methods compared (Supplementary Table 3). Shown here are only the best performing methods that solely use conservation measures (see Supplementary Table 3 for the results of other methods tested). AUC values are provided for each method in parentheses. **b** Precision-recall curves for the same tools and data set

Performance comparisons with methods that rely exclusively on conservation measures, like LIST, are important for the assessment of whether our new conservation measures provide true advantages. Using the ClinVar/ExAC test set, LIST (AUC: 0.888) achieves a substantially higher area under the curve (AUC) value for receiver operating characteristics (ROC) curves than all methods of this type tested, including phyloP_V (AUC: 0.820), SIFT (AUC: 0.818), PROVEAN (AUC: 0.816), and SiPhy^37^ (AUC: 0.810) (Fig. 3a, Supplementary Table 3 and Supplementary Note 1). Importantly, LIST has a strikingly higher precision than the four best performing other methods (phyloP, SIFT, PROVEAN, and SiPhy) at any level of sensitivity (Fig. 3b). Some methods that rely on conservation measures only, such as EVmutation^24^ and LRT^38^, have specific alignment requirements and, thus, score considerably lower numbers of variants. EVmutation for instance, takes co-evolution into account, and thus has higher alignment depth requirements compared to other methods. Also for the subset of variants scored by EVmutation and LRT, respectively, LIST achieves higher AUCs (Supplementary Table 3).

We made several controls to ensure that better predictions by the new measures are not dependent on class definitions. LIST outperforms existing methods independent of the allele frequency used to define common (benign) variants in the ClinVar/ExAC test set (Supplementary Fig. 3, Supplementary Table 4 and Supplementary Note 2). We also controlled for the independence of our findings on the selection of the variants used in optimization and testing (Supplementary Table 5 and Supplementary Note 3). Importantly, we tested LIST’s performance on the additional test sets UniProt/gnomAD and HumVar, which have deleterious and benign variant classes collected using different sources (Supplementary Table 2). LIST continues to have an advantage over all tested methods (Supplementary Tables 6, 7), with the exception of the subset of HumVar variants scored by EVmutation, for which SIFT (AUC: 0.888) and EVmutation (AUC: 0.890) outperform LIST (AUC: 0.885) slightly. As one of the rationales for the development of our new conservation measures is the occurrence of variants in distant species that have gain-of-function and potential oncogenic effects in humans, we also tested LIST on the Cancer test set. This test set has the same benign variants as the UniProt/gnomAD test set, but the deleterious class contains only cancer-associated variants (Supplementary Table 2). LIST also outperforms other methods (Supplementary Fig. 4a, Supplementary Table 8) on this Cancer test set. The comparisons and controls that we carried out demonstrate that the new conservation measures implemented in LIST provide a higher precision in separating benign and deleterious human variants than classical conservation measures implemented in established methods.

Methods that combine conservation measures with features derived from functional genomics studies and/or gene annotations (e.g., Eigen, CADD, DANN, PolyPhen-2, FATHMM-MKL^39^, or fitCons) generally perform better in the prediction of deleterious variants than methods that rely on conservation measures only. We also contrasted LIST’s performance with that of these predictors using the ClinVar/ExAC, UniProt/gnomAD, HumVar, and Cancer test sets. LIST outperforms also these methods on nearly  all these sets (Supplementary Figs. 4b, 5a, and b, Supplementary Tables 3, 4, 6–8), with the exception of the HumVar set, where Eigen achieves a slightly higher AUC than LIST (Supplementary Table 7).

### LIST’s advantages over existing methods

Next, we compared performances for variants that are located in sequence segments of different alignment depth. Most applications exploiting variant frequencies struggle at shallow alignment depth (Supplementary Table 9), therefore, they are less accurate when variants are located in intrinsically disordered protein regions (IDRs)^40^, which are enriched in sequences with low alignment depths (Supplementary Fig. 6). LIST performs better than SIFT and PROVEAN, which we took as representative methods, in evaluating variants located in sequence segments with very low and very high alignment depth (Supplementary Table 10). LIST also does better than all other tested methods when evaluating variants in protein parts predicted to be disordered by ESpritz^41^ or IUPred^42^ (Supplementary Fig. 7 and Supplementary Tables 3, 6–8). This said, all methods perform worse on variants located in IDRs when compared with their performance on all variants. To assess the relative drop in performance for variants in IDR regions, we calculated ${\mathrm{\varphi }}_{{\mathrm{IDR}}} = ({\mathrm{AUC}}_{{\mathrm{ALL}}} - {\mathrm{AUC}}_{{\mathrm{IDR}}})/({\mathrm{AUC}}_{{\mathrm{ALL}}} - 0.5)$. This calculation revealed that the relative performance drop on the ClinVar/ExAC test set is only 14.3% for LIST but 20.0%, 26.1%, 20.7%, 24.4%, 21.4%, and 22.9% for PhyloP_V, SIFT, PROVEAN, SiPhy, GERP++, and phastCons_V, respectively.

**Fig. 4:**
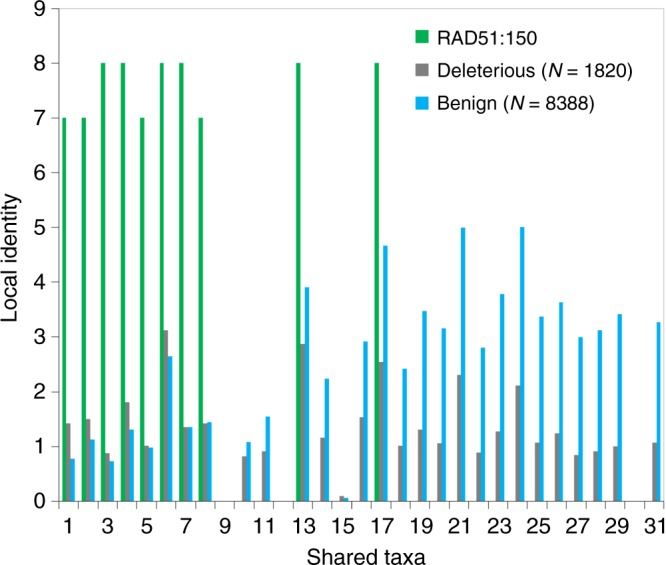
Example illustrating the advantage of new conservation measures. STP for position 150 of protein RAD51 compared with the averaged STPs of benign and deleterious  variants

Finally, we selected an example that showcases the advantage of our new conservation measures. The deleterious variant R150Q of the human recombinase *RAD51* is associated with hereditary breast cancer but predicted by SIFT and PROVEAN to be benign. The false predictions by SIFT and PROVEAN can be attributed to the high frequency of amino-acid Q (17.5%) in the MSA, which is even higher than the frequency of the reference amino acid R (6.1%) (Supplementary Fig. 8a). Interestingly, EVmutation predicts Q to be more benign than the reference amino acid R. LIST, by contrast, scores the R150Q variant in *RAD51* differently. Although VST for Q is 17 (Supplementary Fig. 8b), and, thus, the score of LIST’s first module suggests a neutral to benign character for R150Q (see Fig. 2), most variants differing from the human reference allele R occur in homologs of species that are far from human in the taxonomy tree (Fig. 4), resulting in a high score from the second LIST module and a high overall score, which suggests deleteriousness.

## Discussion

We introduced a new framework and measures for conservation. Algorithmic implementations of these new measures in LIST provide conservation scores that increase precision in the prediction of the deleteriousness of human variants. LIST shows a substantial improvement over methods, such as SIFT or PROVEAN, that rely solely on established conservation measures. Although not exploiting functional genomics data or results from other predictors, LIST also outperforms predictors that use such information including Eigen, CADD, DANN, and PolyPhen-2. This result is particularly remarkable when considering that CADD, for instance, utilizes 10 SVM-linear models trained on 63 distinct features including conservation measures, regulatory, and transcript information as well as scores computed by other predictors such as SIFT and PolyPhen-2. It is important to note that there exists another category of methods, including the predictors FATHMM-weighted^43^ and PON-P2^44^, that estimates the deleteriousness level of each protein and integrates this information with variant deleteriousness scores to improve the overall accuracy across multiple proteins. PON-P2^44^, for example, achieves higher precision in separating deleterious and benign variants^45^ than other methods by utilizing, in addition to conservation and genomic annotations, GO terms to estimate protein level deleteriousness. LIST scores are protein independent and reflect the deleteriousness based on the likelihood of a mutation to alter the molecular functions of the mutated protein regardless of the deleterious level of that protein. Thus, predictors from this additional category and LIST are categorically different and not directly comparable. Furthermore, some existing predictors utilize minor allele frequencies as an input feature^46,47^ and are also not directly comparable to LIST.

We built LIST based on the hypothesis that the taxonomic closeness of species from which sequences in MSAs originate has to be taken into account when assessing conservation. What this specifically means is best explained for the case where an amino-acid matching a human variant of interest is found in the MSA. A human variant for which such a matching amino acid already exists in the reference of another species is more likely to be benign when that species is closely related to human than farther way in the taxonomy tree. In contrast, the human variant is more likely to be deleterious when a matching amino acid is present in homologous proteins of a far-related species. Both of these corollaries, which we demonstrate to be correct, follow the concept that the overall similarity of species influences the phenotypic impact of identical amino acids at given positions in homologous protein.

It can be expected that identical amino acids at a specific sequence position in homologous proteins have similar effects if two species that harbor these homologs are close in the taxonomy tree. As a consequence, if an amino acid has a benign effect in one of these closely related species, thus in its reference sequence, this amino acid is most likely benign in the other species as well. However, identical amino acids at the same sequence position of homologous proteins in two species that are far apart in the taxonomy tree can have very different effects, as interaction partners and regulatory mechanisms are likely to be different for the homologous proteins . The replacement of phosphosite residues by phospho-mimicking aspartic or glutamic acid in lower eukaryotes, prokaryotes, and archea^32^, as mentioned in the introduction, provides a good example for this case. Although potentially detrimental in humans^33^, the replacement of a phosphoswitch by a permanent “on-state”, via phospho-mimicking amino acids, may be tolerated in unicellular organisms because of different cellular regulatory mechanisms in these organisms and/or different selective pressures acting on them. In this context, it is also interesting to note that, based on recent data, it has been suggested that the aggressive behavior of human cancer cells might be the result of atavistic processes that bring back unicellular behavior^48–51^. Whether human variants that have matching amino acids in homologs of far-related, unicellular species contribute to the activation of such atavistic processes in cancer remains to be established. In any case, to provide a first test for the potential relevance of our new measures in the identification of cancer-causing variants, we also evaluated LIST performance on the Cancer test set. We found that LIST indeed achieves a high AUC on this set, in contrast to most of the existing methods that drop significantly in performance on this set. More studies are clearly required to establish whether variants that are deleterious to humans and occur in the sequence of species that are far away from them in the taxonomy tree are indeed more associated with cancer than with other diseases.

In summary, we demonstrate that measures exploiting the taxonomic closeness of species are more effective for the assessment of the deleteriousness of human variants than measures exploiting variant frequency across species. Therefore, we believe that the conservation measures that we introduced will be useful for many applications investigating the in vivo effect of variants that change protein-protein interactions, protein regulation and signaling, or other protein features that are cellular context dependent. Hence, taxonomy-based conservation measures are likely to constitute a more reliable alternative to frequency-based measures for a wide range of applications spanning all biosciences.

## Methods

### Data sets

Our main data sets are based on exome variants that originate from 60,706 individuals, which were identified through high-throughput methods and collected by the Exome Aggregation Consortium^34^ (ExAC). To avoid multi-isoform redundancy, we only used variants that mapped to the SwissProt human protein sequences (downloaded on August 9, 2017). These ExAC variants were mapped to the human reference genome GRCh37, which is superseded by GRCh38. To avoid cases where different predictors make predictions for identical variants based on different sets of underlining sequences, we only used those variants that map to identical regions in both GCRh37 and GCRh38. Variants of the first amino acid M and those involving nonstandard amino acids were excluded for all analyses. We divided the ExAC data and created three sets (optimization sets 1 and 2, ExAC/ClinVar test set) for optimization and initial testing (Supplementary Table 2). To avoid protein level bias^52^ during optimization and testing, we first divided the SwissProt human protein sequences randomly into two equal sets A and B such that sequences in either set have < 50% identity with those in the other set. Variants that map to proteins in set A were used for optimization only (optimization sets 1 and 2), and those that map to proteins in set B were used for testing only (ExAC/ClinVar; Supplementary Table 2). For optimization set 2 and the ExAC/ClinVar test set, variants in ExAC that are marked by ClinVar as pathogenic were placed in the deleterious class and, from the remaining variants, those that are observed in the human population with an allele frequency (ExAC: Adjusted Alt allele frequency in total ExAC samples) ≥1% were considered to be benign and placed in the benign class. The optimization set 2, was used to optimize the hierarchical structure of LIST. The number of variants in optimization set 2 is small (deleterious/benign: 2,146/18,109), especially the number of deleterious variants. Thus, for training of LIST’s individual modules, we generated optimization set 1, where the deleterious class is defined as rare variants with allele frequency in the range of 0.015% to 0.03%, and the benign class as frequent variants with allele frequency ≥ 0.5%. Optimization set 1 contains 24,096 benign and 48,142 deleterious variants. When generating the histograms of VST values and raw frequencies for ExAC variants with deleterious and benign effects (Fig. 2 and Supplementary Fig. 1), we used the Optimization set 2 and excluded variants with alignment depth < 50. For Fig. 2a and Supplementary Fig. 1, we also excluded those variants that do not match any amino acid in the raw MSA (43.4% of the deleterious and 15.1% of the benign variants with alignment depth ≥ 50 did not match any amino acid in MSA). Importantly, the trends shown in Fig. 2a and Supplementary Fig. 1 are reproduced when using the entire ExAC/ClinVar data set. The ExAC/ClinVar test set was used to analyze the performance of LIST and compare it to other methods. LIST scores all variants in this test set (see Supplementary Table 2). However, most methods that we compared LIST’s performance with do not score all variants. Therefore, for each type of analysis presented, we used the maximal number of variants from the ExAC/ClinVar test set that were scored by all methods used in the comparison.

We created two additional test sets (gnomAD/UniProt, and Cancer) and also used the HumVar^53^ data set. For each of these sets, only variants mapping to protein set B were used. In the additional test set gnomAD/UniProt, deleterious variants are those that are marked by UniProt as pathogenic and benign variants are those with an allele frequency ≥ 1% in the gnomAD data set (Alternative allele frequency in the whole gnomAD exome samples) and not marked by UniProt as pathogenic. The Cancer test set is a subset of the gnomAD/UniProt test set. It has the same benign variants as the UniProt/gnomAD test set, but only those that are associated with cancer are labeled as deleterious. Finally, the HumVar test set is the subset of HumVar variants provided by PolyPhen-2^29^ that map to proteins of set B, where deleterious variants include all variants associated with diseases and loss of activity/function, excluding those associated with cancer, and benign variants are those that are frequent (allele frequency ≥ 1%) (Supplementary Table 2). It is important to reiterate that all variants used in optimization map to proteins of set A and, thus, do not overlap with variants used for testing because they all map to proteins of set B. As mentioned for the ExAC/ClinVar test set, most methods that we compared LIST’s performance with do not score all variants. Therefore, we always used the maximal number of variants from each set that was scored by all the methods compared.

### Multiple sequence alignment

We aligned each of the 20,195 human SwissProt sequence to the SwissProt/TrEMBL database (downloaded on 9 August 2017) using blastp^54^ with the “outfmt” 4 to generate multiple sequence alignments. We used an e-value cutoff of 0.01, gap opening penalty of 11 and a gap extension penalty of 2. To avoid scenarios where highly conserved and redundant domains saturate the alignment process, which would leave partially conserved protein regions under-aligned or unaligned, we tried not to limit the number of aligned sequences or the alignment depth. Thus, we set the “num_alignments” and the “num_descriptions” parameters to 150,000. We marked two aligned residues at each side of gaps as boundary residues (BND), which are handled differently by our algorithm as described in the following section. Finally, we filtered-out aligned protein sections with  ≤40% identity to the human query as well as sections shorter than either 70 residues or 70% of the query sequence length; whatever is smaller. We define the alignment depth at position *τ* as the number of sequences with LI ≥ 4 at *τ*.

### Measures and LIST modules

LIST uses two key metrics that we have to define before providing details on the individual modules.

The local identity, LI, of an aligned sequence at *τ* is defined as the number of identical residues between that sequence and the human query in a window size 9 centered at *τ*, excluding the residue at *τ*. Sequences with a residue labeled as BND at position *τ* are assigned a LI of zero. The window size of 9 was learned, using a grid search, to maximize LIST’s AUC for predictions of variants in the optimization set 1.

Shared taxa ST is defined as the number of edges in the taxonomy tree that are shared between human and other species (Fig. 1a).

LIST is constructed hierarchically^35,36^ (Supplementary Fig. 2) from the three modules PVM, PM, and AM. All modules have been designed such that their output scores correlate positively with deleteriousness, i.e., higher scores indicate higher likelihood for deleteriousness.

Position variant module (PVM): PVM exploits the contrast in the variant shared taxa VST between benign and deleterious variants shown in (Fig. 2a). For any given variant *x* at position *τ*, the PVM_τ,x_ score is computed as

1 $${\mathrm{PVM}}_{{\mathrm{\tau ,x}}} = \left\{ {\begin{array}{*{20}{c}} {1 - \frac{{{\mathrm{VST}}_{{\mathrm{\tau }},{\mathrm{x}}}}}{{31}},{\mathrm{LI}}_{{\mathrm{max}}} \ge \alpha } \\ {1,{\mathrm{LI}}_{{\mathrm{max}}} < \alpha } \end{array}} \right.$$

The variant shared taxa VST_τ,x_ is the ST value of the sequence of highest LI to the query and with amino acid *x* at *τ*. We assume that the sequence with the highest local identity around *τ* comes from a homologous gene. To guarantee a minimal level of homology, i.e. functional relation, only sequences with LI_max_ *>* *α* are considered. The cutoff *α* = 4 was obtained by maximizing PVM’s AUC value using the optimization set 1. If no matching amino acid is found, LI is set to 0 and PVM_τ,x_ to 1. If the highest LI is shared by several sequences, to break the tie, we use the highest section identity, SI, to identify the closest homolog. SI of a given sequence is defined as the number of residues that are identical between the human query and the section of this sequence that harbors position *τ* and is continually aligned in the blastp output. Residues labeled with BND are considered as mismatches in the calculation of SI. If multiple sequences share the same highest SI (and the same highest LI), then we select the highest ST from this pool of sequences.

Position module 1 (PM1): PM1_τ_ is the average of the PVM_τ,x_ scores of all possible amino acids *x* at position *τ* excluding the amino acid of the reference

2 $${\mathrm{PM}}1_{\mathrm{\tau }} = \frac{{\mathop {\sum }\nolimits_{{\mathrm{x}} \ne {\mathrm{ref}}}^{20} {\mathrm{PVM}}_{{\mathrm{\tau }},{\mathrm{x}}}}}{{19}}$$

Position module 2 (PM2): PM2 exploits the contrast between the average STPs (described earlier) of deleterious and benign variants shown in Fig. 2b. In this figure, the blue (gray) column at each ST value represents the averaged maximum LI values of all benign (pathogenic) variants associated with that shared taxa. Averaged STPs reveal that benign variants have higher LIs at higher STs compared with deleterious ones. Thus, a simple linear classifier is used to exploit this contrast.

First, we computed the average log STPs of deleterious and benign variants:

3 $${\mathrm{LSTP}}_{{\mathrm{deleterious}},{\mathrm{st}}} = \frac{{\mathop {\sum }\nolimits_{{\mathrm{\tau }} = {\mathrm{deleterious}}} {\mathrm{LSTP}}_{{\mathrm{\tau }},{\mathrm{st}}}}}{{{\mathrm{N}}_{{\mathrm{deleterious}}}}}$$

4 $${\mathrm{LSTP}}_{{\mathrm{benign}},{\mathrm{st}}} = \frac{{\mathop {\sum }\nolimits_{{\mathrm{\tau }} = {\mathrm{benign}}} {\mathrm{LSTP}}_{{\mathrm{\tau }},{\mathrm{st}}}}}{{{\mathrm{N}}_{{\mathrm{benign}}}}}$$

Where ${\mathrm{LSTP}}_{{\mathrm{\tau }},{\mathrm{st}}} = \log _{10}\left( {1 + {\mathrm{STP}}_{{\mathrm{\tau }},{\mathrm{st}}}} \right)$, and N_deleterious_ (N_benign_) is the number of protein positions in the optimization set 1 with deleterious and no benign variants (benign and no deleterious variants).

Then, for each ST value, we computed the center of these two profiles and the span between them:

5 $${\mathrm{LSTP}}_{{\mathrm{center}},{\mathrm{st}}} = \frac{{{\mathrm{LSTP}}_{{\mathrm{deleterious}},{\mathrm{st}}} + {\mathrm{LSTP}}_{{\mathrm{benign}},{\mathrm{st}}}}}{2}$$

6 $${\mathrm{LSTP}}_{{\mathrm{span}},{\mathrm{st}}} = {\mathrm{LSTP}}_{{\mathrm{deleterious}},{\mathrm{st}}} - {\mathrm{LSTP}}_{{\mathrm{benign}},{\mathrm{st}}}$$

And finally, the PM2_τ_ score for any sequence location τ is defined as

7 $${\mathrm{PM}}2_{\mathrm{\tau }} = \frac{{\mathop {\sum }\nolimits_{{\mathrm{st}} = 1}^{31} \left( {{\mathrm{LSTP}}_{{\mathrm{\tau }},{\mathrm{st}}} - {\mathrm{LSTP}}_{{\mathrm{center}},{\mathrm{st}}}} \right) \ast {\mathrm{LSTP}}_{{\mathrm{span}},{\mathrm{st}}}}}{{31}}$$

Figure 2b shows that the span between the averaged local identities (LI) of benign and deleterious variants at ST=7, for example, is close to zero. Therefore, at ST=7, the STP value has no contribution to the PM2 score. In contrast, the span calculated at ST=27 is large, and the LI value at that ST has a high impact on the PM2 score.

The amino acid module (AM): Benign variants are more likely to replace reference amino acids with new ones that have a similar physiochemical property (swap-ability) when compared with deleterious variants. The AM scores variants solely based on the swap-ability between reference and observed amino acids. We estimated amino-acid swap-ability in the general human population based on counts in the optimization set 1. We constructed the probability matrices PR (PC) of rare (common) variants of the optimization set 1 by counting them and then normalizing over *r*:

8 $${\mathrm{PR}}_{{\mathrm{r}},{\mathrm{x}}} = \frac{{{\mathrm{CR}}_{{\mathrm{r}},{\mathrm{x}}}}}{{\mathop {\sum }\nolimits_{{\mathrm{x}_{j}},_{{\mathrm{j}} \ne {\mathrm{r}}}}^{19} {\mathrm{CR}}_{{\mathrm{r}},{\mathrm{x}_{j}}}}}$$

9 $${\mathrm{PC}}_{{\mathrm{r}},{\mathrm{x}}} = \frac{{{\mathrm{CC}}_{{\mathrm{r}},{\mathrm{x}}}}}{{\mathop {\sum }\nolimits_{{\mathrm{x}_{j}},_{{\mathrm{j}} \ne {\mathrm{r}}}}^{19} {\mathrm{CC}}_{{\mathrm{r}},{\mathrm{x}_{j}}}}}$$

Where *r* is the reference amino acid and *x* is the observed one. PR_r,x_ (PC_r,x_) is the probability of observing rare (common) *r* to *x* changes. CR_r,x_ (CC_r,x_) is the count of rare (common) *r* to *x* changes.

Using PR_r,x_ and PC_r,x_, the AM score is then defined as:

10 $${\mathrm{AM}}_{{\mathrm{r}},{\mathrm{x}}} = \frac{{{\mathrm{PR}}_{{\mathrm{r}},{\mathrm{x}}}}}{{\left( {{\mathrm{PR}}_{{\mathrm{r}},{\mathrm{x}}} + {\mathrm{PC}}_{{\mathrm{r}},{\mathrm{x}}}} \right)}}$$

### Compensating for alignment depth

When comparing LIST’s performance to that of SIFT and PROVEAN for variants of the optimization set 2 that were binned according to the alignment depth at their locations (Supplementary Table 9), it became clear that LIST outperforms both predictors at all alignment depths. However, when LIST was used to predict variants at locations covering the entire spectrum of alignment depths, LIST performed well but not as well as for the predictions of variants that were binned according to alignment depths. The reason for this became obvious when analyzing the median scores of each module for the different bins. PVM and PM1 median scores were roughly constant across the different bins, whereas PM2 median scores shifted toward smaller and even negative values, thus correlating inversely with alignment depth (Supplementary Table 9). This shift in scores has no significant impact on predictions made only for variants within a specific bin because each bin spans a small range of the alignment depth. However, it affects predictions across all alignment depths. Furthermore, Supplementary Table 9 revealed that variants in regions of higher alignment depth are more likely to be deleterious compared to those in lower alignment depth. We are using weighted Bayes rule to integrate the scores of LIST modules hierarchically into LIST final score. In theory, Bayes rule computes the probability of an event by combining different, independent, probabilities of that event. In our case, raw scores computed by individual LIST modules are not real probabilities of the deleteriousness of variants, and dependencies between these scores are difficult to estimate. Consequently, we had to process raw scores before integrating them so that they reflect, as much as possible, real probabilities.

We undertook two processing steps. First, we rescaled PM2 scores to factor out alignment depth. Specifically, we computed the range of PM2 scores at each alignment depth using the optimization set 2 and then, when evaluating query sequences, shifted PM2 score appropriately according to the precomputed range associated with its alignment depth (Supplementary Note 4). Second, we scaled the scores of all modules to reflect the fact that variants at higher alignment depth are more likely to be deleterious compared to those at lower alignment depth, i.e., have higher probability of been deleterious. Specifically, we used the optimization set 2 to estimate the probability of deleterious variants for each alignment depth, and then, to reflect real probabilities, multiplied each of the PVM, PM1, and PM2 scores by the precomputed probability of deleterious mutations associated with its alignment depth (Supplementary Note 4). Importantly, the prediction performance for variants within specific bins changed only marginally for most bins as a result of this scaling, highlighting that compensating for alignment depth helped mainly in making scores consistent for predictions across all alignment depths.

Note that many tools do not score mutations at shallow alignment depth. LIST assigns unscaled, median scores from PVM, PM1, and PM2 to variants at positions with alignment depth < 3 and then compensates this scores for alignment depth (first and second explained above). This alignment depth cutoff value of 3 was learned to maximize AUC using the optimization set 2. Consequently, LIST scores all variants regardless of the alignment depth.

### Optimizing LIST hierarchical structure

We made the following two assumptions:

First, we assumed that, once compensated for alignment depth, each module’s scores can be loosely considered probabilities after being rescaled to the range [0+*C*, 1−*C*] and then weighted:

11 $${\mathrm{rescaled}}\_{\mathrm{score}} = C + \left( {\left( {1 - 2C} \right) \ast \frac{{{\mathrm{score}} - {\mathrm{min}}}}{{\max - {\mathrm{min}}}}} \right)$$

where min and max are the minimum and maximum scores for each module observed in the optimization set 2. *C* = 0.2 was used to prevent extreme values from dominating the final outcome. A weight (*ω* ∈ [0.1,1]) is used to account for the relative prediction accuracy of each module, and the weighted scores were calculated as:

12 $${\mathrm{weighted}}\_{\mathrm{score}} = 0.5 + \left( {\left( {{\mathrm{rescaled}}\_{\mathrm{score}} - 0.5} \right) \ast \omega } \right)$$

Weights *ω* were learned using a grid search on optimization set 2 to maximize LIST’s AUC, such that modules with higher accuracy are assigned higher *ω* values. Low *ω* values produce weighted scores that reflect high uncertainty, i.e., probabilities near 50%, whereas weighted scores resulting from higher *ω* values have more impact on the final score that is generated by combining weighted scores using Bayes rule^35,36^.

Second, the output scores that are generated when combining weighted scores using Bayes rule are likely to be skewed away from the center because the different input scores are not completely independent. Thus, in order to use it as an input probability to the next hierarchical level, these scores are redistributed to fit a normal distribution centered at Bayes rule identity element 0.5, *N*(*μ* = 0.5, *σ*^2^ = 0.01) and bounded by the range [0+*C*, 1−*C*] (Supplementary Note 5, Supplementary Figs. 9 and 10).

We redistribute LIST’s output scores to fit a uniform distribution (i.e., rank score), which, we believe, makes the interpretation of these scores simpler. We learned the redistribution function from optimization set 2. The final ROC curves representing the performances of each of LIST three sub-modules are shown in Supplementary Fig. 11.

In the practical use of methods like LIST, variants of interest are scored, and the subset of variants with highest scores are prioritized for experimental testing or other ways of validation. Therefore, it is important that predictors score all variants fairly and with as little bias as possible. Otherwise, the scores of training variants will dominate and overshadow those that are novel. The hierarchical learning approach applied here enables the use of simple learning tools (linear models) that pose limited risk of over-scoring variants used in optimization. Indeed, our results demonstrate that LIST poses virtually no bias in scoring variants used in its optimization over those that are used for testing (see Supplementary Note 3, Supplementary Table 5).

In order to provide access to this new tool, we set up a server with precomputed LIST predictions of all possible variants in SwissProt human protein sequences: http://list.msl.ubc.ca/ (Supplementary Fig. 12).

### Reporting summary

Further information on experimental design is available in the Nature Research Reporting Summary linked to this article.

## Supplementary information

Supplementary Information

Reporting Summary

## Data Availability

The data sets generated during the current study are available in the UBC Michael Smith Laboratories Dataverse repository: 10.5683/SP2/BE6AEA Data associated with figures can be found in the source data file: https://doi.org/10.5683/SP2/3OU15O LIST can be found at http://list.msl.ubc.ca/ Protein sets used in the optimization and benchmarking of LIST are available at: https://gsponerlab.msl.ubc.ca/software/list/ SwissProt/TrEMBL protein sequences are available from UniProt: https://www.uniprot.org/ Taxonomy data are available from NCBI: https://www.ncbi.nlm.nih.gov/guide/taxonomy/ ExAC and gnomAD allele frequencies and prediction scores for SIFT, PROVEAN, phyloP, SiPhy, GERP++, phastCons, LRT, Eigen, CADD, Fathmm-MKL, DANN, MutationTaster, Polyphen-2, MutationAssessor, GenoCanyon, and fitCons were downloaded from dbNSFPv3.5: https://sites.google.com/site/jpopgen/dbNSFP/ EVmutation scores were downloaded from: https://marks.hms.harvard.edu/evmutation/ All other relevant data are available upon request.

## Code availability

LIST’s code is available at: https://github.com/NawarMalhis/LIST.
