# Supplementary material for: Improved measures for evolutionary conservation that exploit taxonomy distances
Source: Nat Commun. 2019 Apr 5;10:1556. doi: 10.1038/s41467-019-09583-2 (PMC6450959; doi:10.1038/s41467-019-09583-2)
Supplement: Supplementary file 1 — Supplementary Information [file 41467_2019_9583_MOESM1_ESM.pdf]

Improved measures for evolutionary conservation that exploit taxonomy distances

Nawar Malhis et al.

Supplementary Figures

Figure 1:

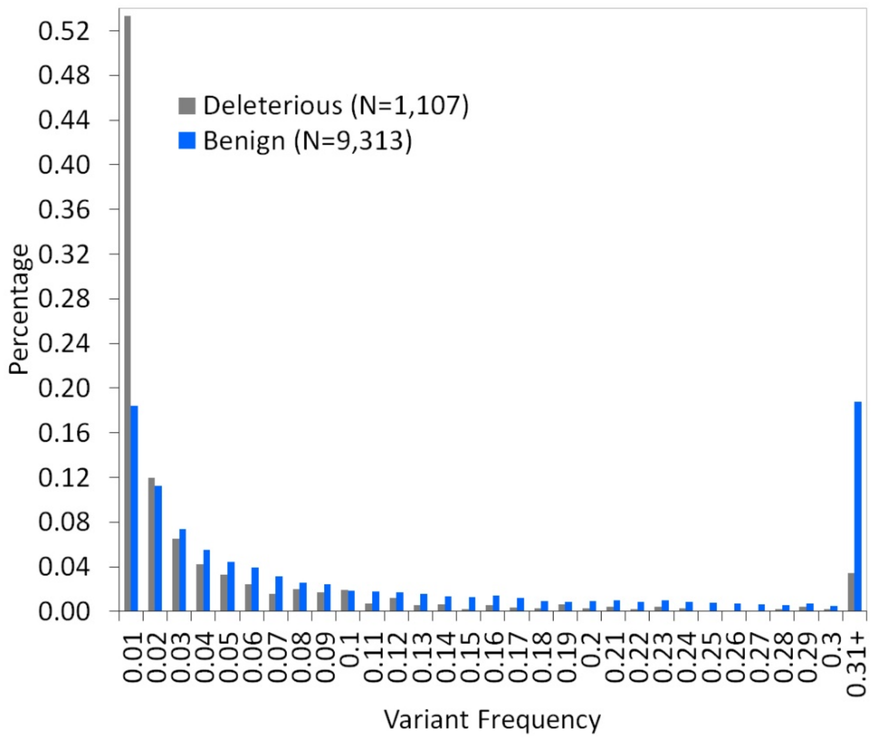

Figure 1: Distribution of frequencies in MSA for deleterious and benign variants that have a matching amino acid in the raw MSA and an alignment depth greater than 50 (see methods for details on data). Only variants with a matching amino acid in the MSA have been selected to allow for a direct comparison with the VST measure.

Figure 2:

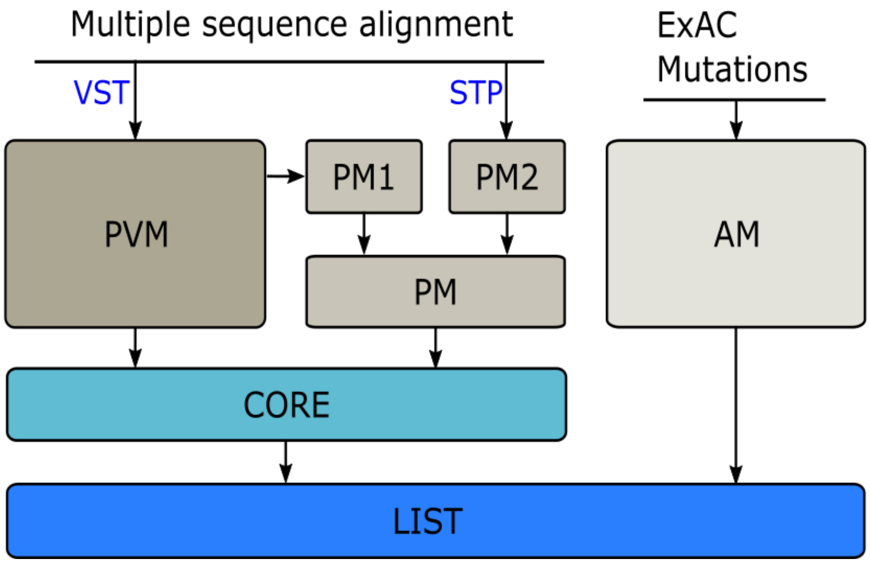

Figure 2: Overview of LIST's hierarchical module structure. Two modules (PVM and PM) generate scores based on shared taxa (ST) and local identity (LI) extracted from MSAs. The position variant module PVM computes scores based on the occurrence of a specific variant at a specific position and relies solely on variant shared taxa VST, while the second, position module PM, computes scores for specific positions independent of the variant. PM utilizes VST with its PM1 module and the shared taxa profile, STP, in its PM2 module. PVM and PM are combined in the CORE module of LIST. The CORE module is complemented with the amino acid module AM, which computes scores based on the probabilities of amino acid substitutions among variants that are rare and common, respectively, in the human population.

Figure 3:

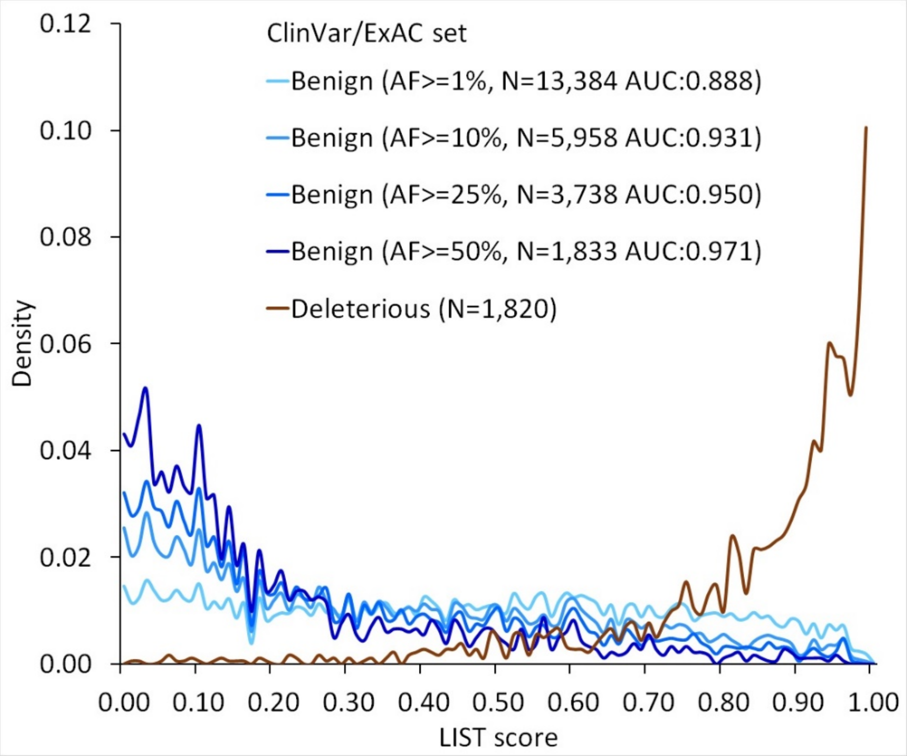

Figure 3: LIST exhibits higher separation power when using higher allele frequency cut-off values to define benign variants.

LIST scores for deleterious (red) and benign variants (shades of blue) in the ClinVar/ExAC test set. Different allele frequency cut-off values were used to define the benign (common) variant set. The number of benign variants N as well as the AUC values for LIST predictions at varying allele frequency cut-offs are provided in parentheses.

Figure 4:

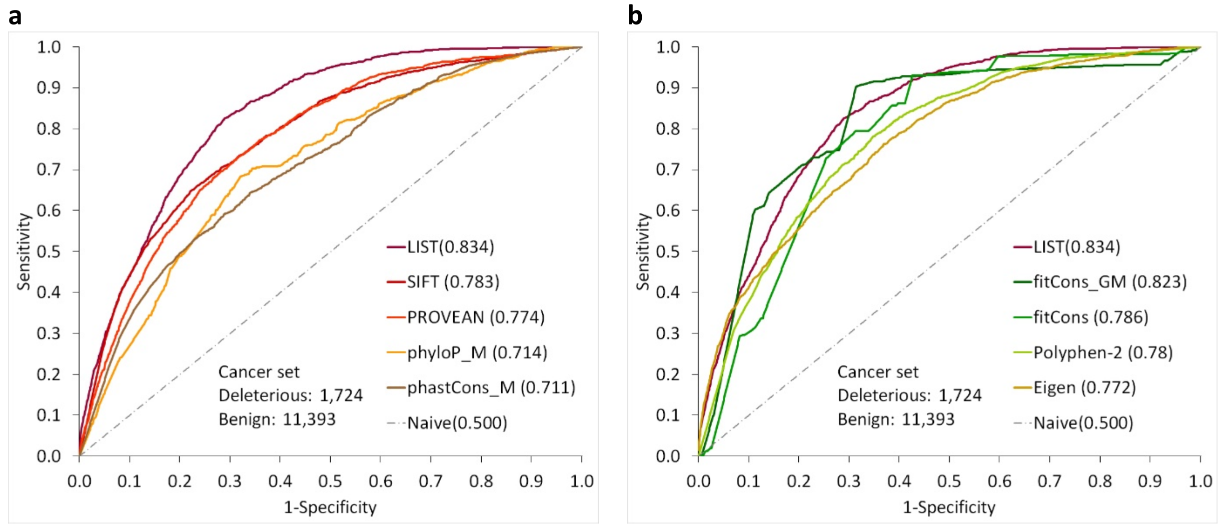

Figure 4: ROC curves for the Cancer test set. We used all variants from the Cancer test set that are scored by all methods compared (intersection). See Supplementary Table 8 for AUCs of all methods compared. ROC curves are shown for LIST and the four best other methods that rely on conservation only (a) or use additional data sources (b). AUC values are provided in the parentheses next to each method's name.

Figure 5:

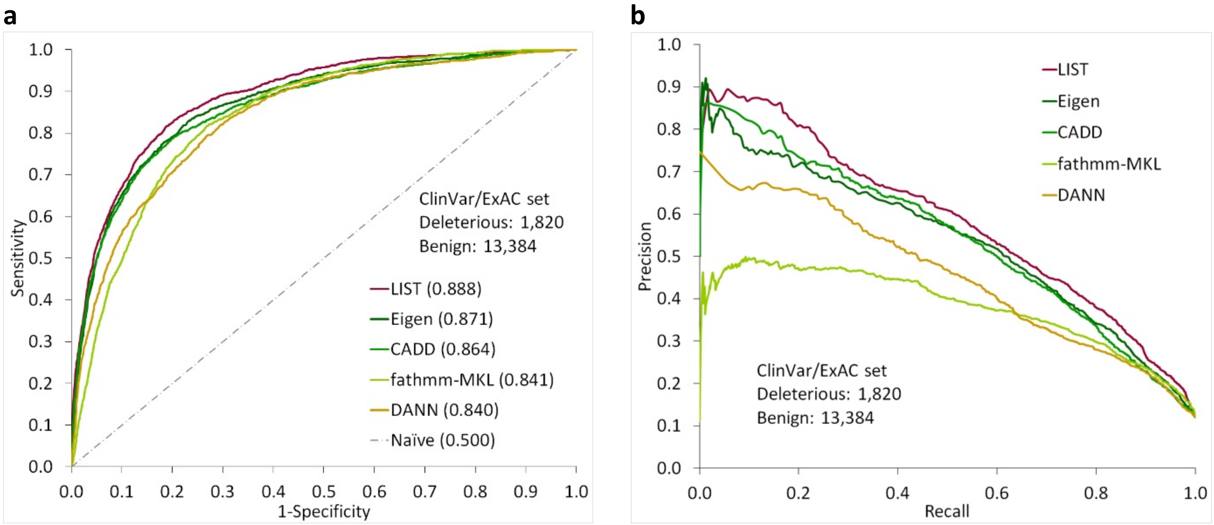

Figure 5: Comparison with methods that combine conservation measures with features derived from functional genomics studies and/or gene annotations using the ExAC/ClinVar test set. We used all variants from the ExAC/ClinVar test set that are scored by all methods compared (intersection). See Supplementary Table 3 for AUCs of all methods compared. ROC curves (a) and precision recall curves (b) contrasting LIST with the four best other methods of this type. AUC values are provided in the parentheses next to each method's name in a.

Figure 6:

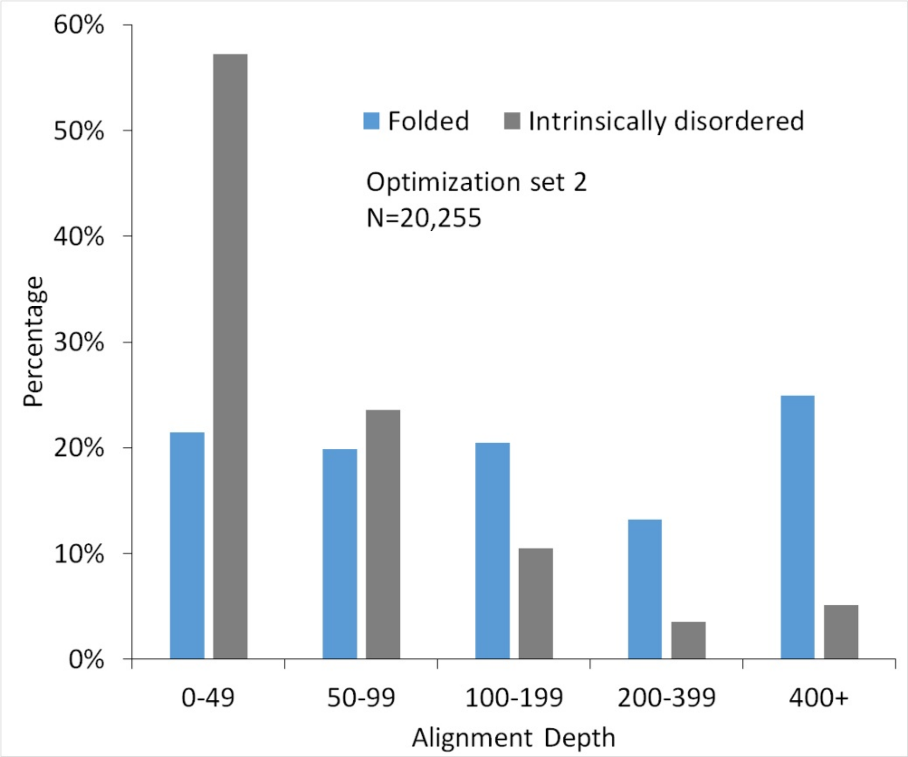

Figure 6: Percentage of residues predicted to be folded or intrinsically disordered (ESpritz or IUpred predictions) in sequences that are binned according to their alignment depth.

Figure 7:

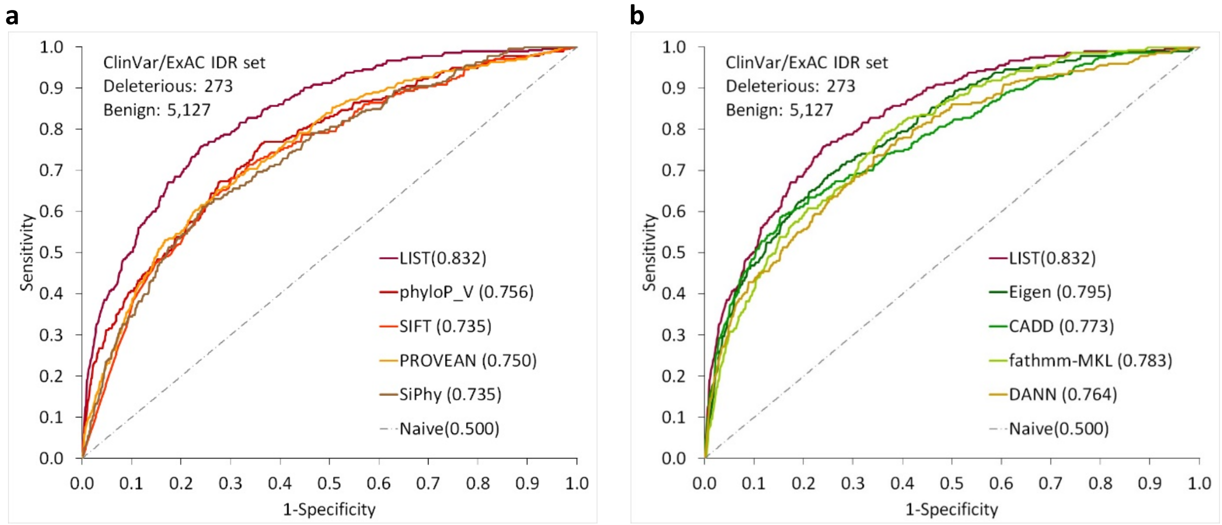

Figure 7: ROC curves for variants from the ClinVar/ExAC test set that are located in regions identified by ESpritz or IUpred as in intrinsically disordered (IDR) and scored by all methods compared (intersection). See Supplementary Table 3 for AUCs of all methods compared. Here, LIST's performance is contrasted with that of the four best other methods that use conservation measures only (a) or combine conservation measures with features derived from functional genomics studies and/or gene annotations (b). AUC values are provided in the parentheses next to each method's name.

Figure 8:

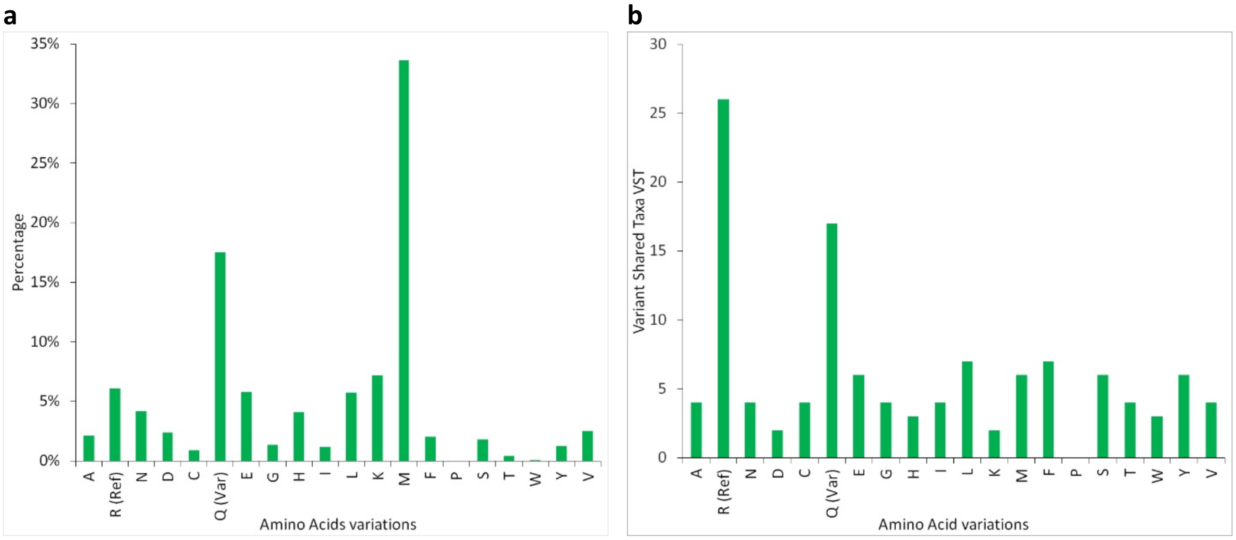

Figure 8: Measures involved in scoring of the variant R150Q of the human DNA repair protein RAD51. (a) Variant frequencies (normalized to percentages of overall occurence) observed in the raw MSA at position 150 and (b) VST values of these variants.

Figure 9:

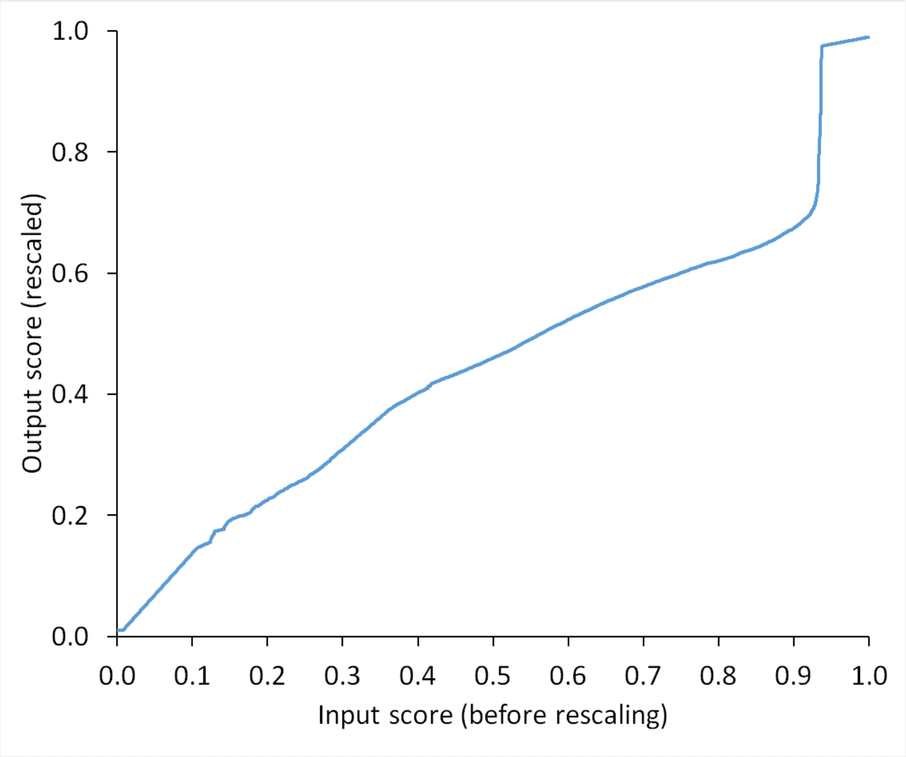

Figure 9: Mapping array learned from the PM scores on the optimization set 2. The horizontal axis is the MAPPING array index divided by its size, which is the input PM score before rescaling (unknown distribution), and the vertical axis is the value of the MAPPING array cell at that index, which is the output rescaled PM score.

Figure 10:

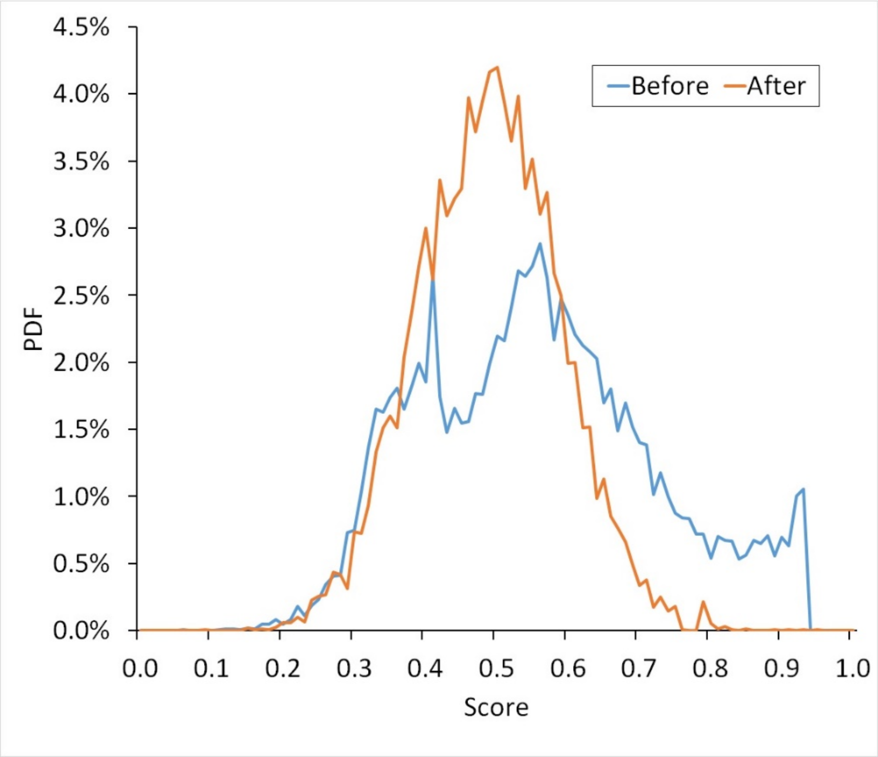

Figure 10: Probability density function of the benchmarking ClinVar/ExAC PM scores before and after redistribution.

Figure 11:

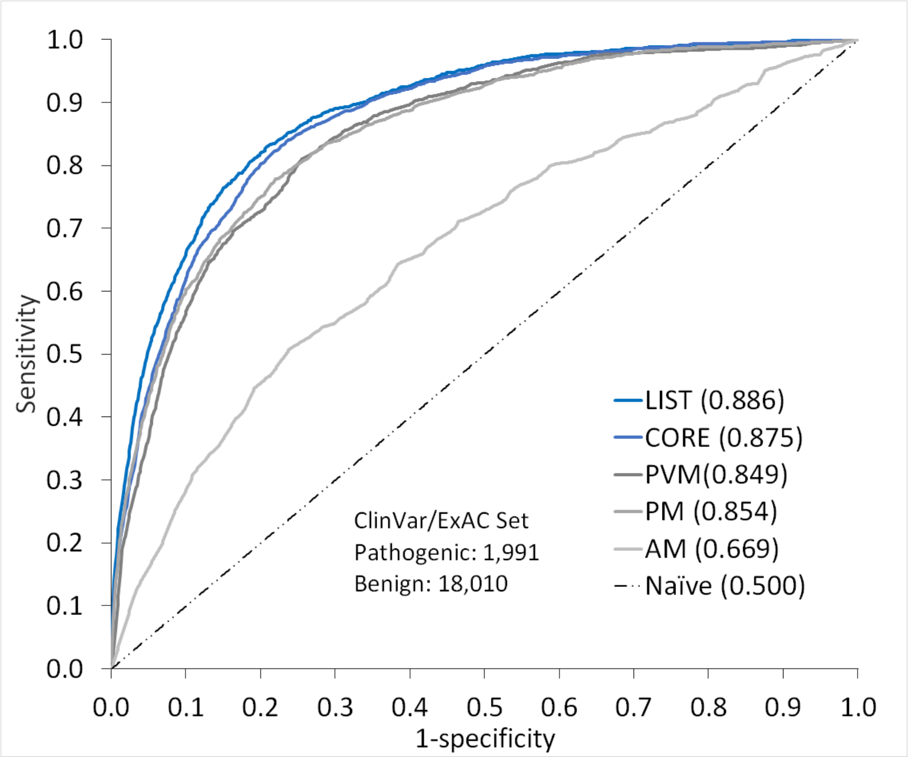

Figure 11: Receiver operating characteristics (ROC) curves of each of the LIST modules using the ClinVar/ExAC benchmarking set. All variants in the ClinVar/ExAC test set are scored by LIST (compare to Supplementary Table 2). Values for the area under the ROC curve (AUC) are provided for each module, CORE and LIST.

Figure 12

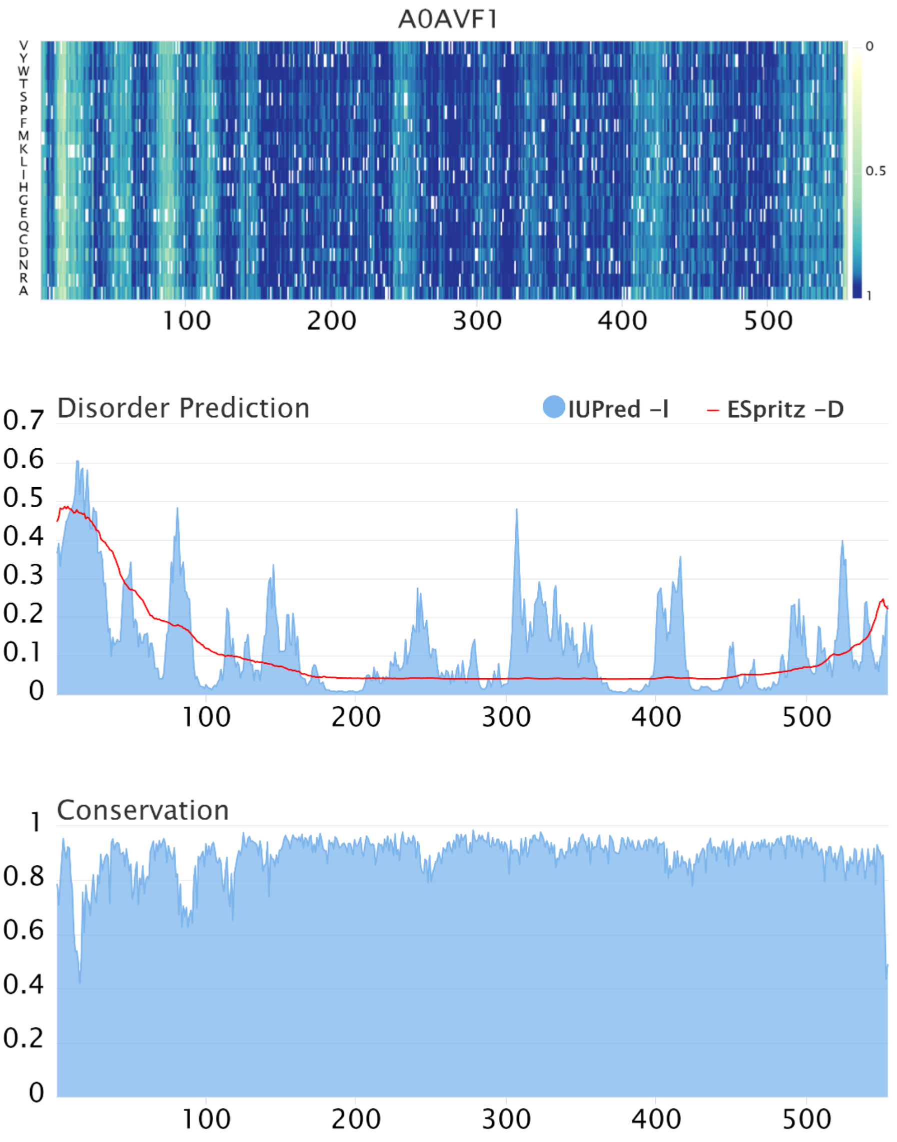

Figure 12: "Screenshot" of the LIST server output for human protein A0AVF1 (IFT56_HUMAN) Top, heat-map matrix representing the level of deleteriousness of all possible variants at each sequence position. Middle, disorder predictions made by IUPred and ESpritz. Bottom, conservation prediction provides the average LIST deleteriousness score for all possible variants at each position.

Supplementary Tables

Table 1: Human taxonomy lineage. The human Taxonomy lineage downloaded from https://www.uniprot.org/ with the associated shared taxa (ST) values.

Shared Taxa	Taxonomy lineage
31	Homo sapiens
30	Homo
29	Homininae
28	Hominidae
27	Hominoidea
26	Catarrhini
25	Simiiformes
24	Haplorrhini
23	Primates
22	Euarchontoglires
21	Boreoeutheria
20	Eutheria
19	Theria
18	Mammalia
17	Amniota
16	Tetrapoda
15	Dipnotetrapodomorpha
14	Sarcopterygii
13	Euteleostomi
12	Teleostomi
11	Gnathostomata
10	Vertebrata
9	Craniata
8	Chordata
7	Deuterostomia
6	Bilateria
5	Eumetazoa
4	Metazoa
3	Opisthokonta
2	Eukaryota
1	cellular organisms

Table 2: Data sets overview.

Optimization and testing sets: We divided the SwissProt human protein sequences randomly into two equal sets A and B. Variants that map to proteins in set A were used for optimization only (optimization sets 1 and 2) and those that map to proteins in set B for testing only. Definitions provide the criteria for how variants were selected. Count provides the total number of variants in each class. It is important to note that the actual number of variants that are used for comparisons or analyses are often smaller because methods other than LIST do not score all variants and/or some of their scores are not deposited in the dbNSFPv3.5[22] database. ExAC_AF is the adjusted alternative allele frequency in total ExAC samples, and gnomAD_AF is the alternative allele frequency in the whole gnomAD exome samples.

**Set Name**	**Protein set**	**Benign**		**Deleterious**	
		Definition	Count	Definition	Count
**Optimization 1**	A	ExAC_AF ≥0.5%	24,096	0.015%≤ExAC_AF ≤0.03%	48,142
**Optimization 2**	A	ExAC_AF ≥1%	18,109	ClinVar pathogenic annotation & ExAC_AF >0	2,146
**ClinVar/ExAC**	B	ExAC_AF ≥1%	18,010	ClinVar pathogenic annotation & ExAC_AF >0	1,991
**UniProt/gnomAD**	B	gnomAD_AF ≥1%	13,926	UniProt pathogenic annotation	14,554
**Cancer**	B	gnomAD_AF ≥1%	13,926	UniProt pathogenic annotation and cancer annotation	2,705
**HumVar**	B	AF ≥1%	10,450	Disease annotations except cancer	11,785

Table 3: Contrasting AUC values using the ClinVar/ExAC test set.

(a) AUC values for LIST and other methods that rely solely on conservation measures. These methods include PhyloP Vertebrata (phyloP_V), SIFT, PROVEAN, SiPhy, GERP++, phastCons Vertebrata (phastCons_V), PhyloP Mammalia (phyloP_M) and phastCons Mammalia (phastCons_M), EVmutation (EVM) and LRT. The first column (ALL) provides the AUCs for all benign and deleterious variants (number provided in the blue top section of the table) that are scored by all methods compared (excluding LRT and EVM). In columns LRT and EVM, we intersect the ALL set with the set of variants that are scored by LRT and EVmutation, respectively. The IDR column contains the AUC for the subset of variants in ALL that are identified by ESpritz or IUpred to be located in intrinsically disordered protein regions. The highest AUCs achieved are highlighted in bold.

**Benign count**	**13,384**	**11,252**	**3,484**	**5,127**
**Deleterious count**	1,820	1,773	1,076	273
	**ALL**	**LRT**	**EVM**	**IDR**
**LIST**	**0.888**	**0.881**	**0.911**	**0.832**
**phyloP_V**	0.820	0.810	0.839	0.756
**SIFT**	0.818	0.819	0.850	0.735
**PROVEAN**	0.816	0.820	0.819	0.750
**SiPhy**	0.810	0.799	0.825	0.735
**GERP++_RS**	0.781	0.766	0.799	0.721
**phastCons_V**	0.778	0.762	0.798	0.714
**phyloP_M**	0.744	0.725	0.778	0.698
**phastCons_M**	0.724	0.702	0.737	0.687
**EVmutation**			0.857	
**LRT**		0.805		

(b) AUC values for LIST and other methods that rely not only on conservation measures. These methods include Eigen, CADD, Fathmm-MKL, DANN, MutationTaster, Polyphen-2, MutationAssessor, GenoCanyon, fitCons HUVEC, fitCons H1-hESC, fitCons integrated (fitCons) and fitCons GM12878.

**Benign count**	**13,384**	**5,127**
**Deleterious count**	1,820	273
	**ALL**	**IDR**
**LIST**	**0.888**	**0.832**
**Eigen**	0.871	0.795
**CADD**	0.864	0.773
**fathmm-MKL**	0.841	0.783
**DANN**	0.840	0.764
**MutationTaster**	0.836	0.764
**Polyphen2**	0.835	0.746
**MutationAssessor**	0.826	0.756
**GenoCanyon**	0.729	0.688
**fitCons_HUVEC**	0.575	0.539
**fitCons_H1-hESC**	0.572	0.546
**fitCons**	0.562	0.544
**fitCons_GM**	0.555	0.525

Table 4: Comparison of prediction performance using different allele frequency cut-offs in the definition of the benign class.

AUC values for the predictions of variants in the in the ClinVar/ExAC test set using different allele cut-off to define benign variants. The cut-offs used are indicted as well as the number of benign variants that result from the use of these cut-offs. The highest AUCs achieved are highlighted in bold. For the remaining of this paper, all results are for the case where the benign class contains those common variants with an AF ≥ 1%.

(a) AUC values of methods that rely solely on conservation measures

**Benign count**	**13,384**	**5,958**	**3,738**	**1,833**
**Deleterious count**	1,820	1,820	1,820	1,820
**Benign cut-off Frequency**	**1%**	**10%**	**25%**	**50%**
**LIST**	**0.888**	**0.931**	**0.950**	**0.971**
**phyloP_V**	0.820	0.850	0.858	0.867
**SIFT**	0.818	0.865	0.893	0.930
**PROVEAN**	0.816	0.858	0.882	0.919
**SiPhy**	0.810	0.839	0.848	0.859
**GERP++_RS**	0.781	0.809	0.812	0.808
**phastCons_V**	0.778	0.808	0.815	0.818
**phyloP_M**	0.744	0.804	0.833	0.872
**phastCons_M**	0.724	0.762	0.773	0.781

(b) AUC values for LIST and other methods that rely not only on conservation measures.

**Benign count**	**13,384**	**5,958**	**3,738**	**1,833**
**Deleterious count**	1,820	1,820	1,820	1,820
**Benign cut-off Frequency**	**1%**	**10%**	**25%**	**50%**
**LIST**	**0.888**	**0.931**	**0.950**	**0.971**
**Eigen**	0.871	0.908	0.923	0.947
**CADD**	0.864	0.900	0.916	0.938
**fathmm-MKL**	0.841	0.885	0.905	0.933
**DANN**	0.840	0.883	0.903	0.935
**MutationTaster**	0.836	0.874	0.883	0.902
**Polyphen2_HD**	0.835	0.879	0.903	0.939
**MutationAssessor**	0.826	0.865	0.889	0.924
**GenoCanyon**	0.729	0.751	0.752	0.753
**fitCons_HUVEC**	0.575	0.588	0.592	0.598
**fitCons_H1-hESC**	0.572	0.586	0.592	0.590
**fitCons**	0.562	0.577	0.581	0.577
**fitCons_GM**	0.555	0.566	0.570	0.574

Table 5: Controls for the influence of the optimization process on scoring.

(a) To assess the influence of the optimization set Y ∈ {A, B} on LIST output scores, we evaluated the performance of LIST on each set X ∈ {A, B}, $AUC_{X/Y}$, twice; once for X≠Y and then for X=Y. Then we computed the effect of optimization for each set $\Delta\ AUC1_X$ as $AUC_{X/X}$ - $AUC_{X/Y}$. On average, the influence of optimization on LIST (Δ AUC1) is small: $\Delta\ AUC1 = (\Delta\ AUC1_A + \Delta\ AUC1_B) / 2 = 0.0043$.

Testing data (X)	Optimization data (Y)	$AUC_{X/Y}$	$\Delta\ AUC1_X$
**B**	A	0.8862	-0.0021
**B**	B	0.8841	
**A**	A	0.8804	0.0065
**A**	B	0.8739	

(b) We can also see from panel (a) that there is a small, but noticeable, difference in LIST performance using set B vs A. This is likely because deleterious and benign variants in the B set are slightly more separable than those in the A set. We can approximately estimate this difference for all the tools as: $\Delta AUC2 = AUC_B - AUC_A$.

**Benign count**	**13,384**	**13,659**	
**Deleterious count**	1,820	1,949	
	**AUC_B**	**AUC_A**	**Δ AUC2**
**fitCons_HUVEC**	0.5752	0.5495	0.0256
**DANN**	0.8398	0.8265	0.0134
**phyloP_M**	0.7437	0.7328	0.0109
**SIFT**	0.8180	0.8096	0.0084
**GenoCanyon**	0.7293	0.7230	0.0062
**LIST***	0.8879	0.8822	0.0057
**phastCons_M**	0.7243	0.7186	0.0057
**MutationAsse**	0.8256	0.8204	0.0051
**GERP++**	0.7815	0.7767	0.0047
**CADD**	0.8638	0.8594	0.0044
**phastCons_V**	0.7780	0.7748	0.0032
**Eigen**	0.8714	0.8688	0.0026
**fathmm-MKL**	0.8413	0.8391	0.0022
**Polyphen2**	0.8345	0.8329	0.0016
**SiPhy_29way**	0.8103	0.8097	0.0005
**MutationTast**	0.8363	0.8361	0.0002
**PROVEAN**	0.8158	0.8159	-0.0001
**fitCons_GM**	0.5555	0.5565	-0.0010
**phyloP_V**	0.8205	0.8226	-0.0021
**fitCons_H1-hESC**	0.5718	0.5802	-0.0084
**fitCons**	0.5620	0.5733	-0.0112

* LIST has been optimized on variants mapped to proteins from set A and tested on those mapped to proteins from set B. In contrast, we are not separating training and testing data for other tools. Thus, to be more accurate in estimating Δ AUC2 for LIST, one can subtract the average of the two $AUC_{X/Y}$ values for each testing set provided in panel a, resulting in a Δ AUC2 for LIST of 0.0080.

Table 6: Contrasting AUC values using the UniProt/gnomAD test set.

(a) AUC values for LIST and other methods that rely solely on conservation measures. These methods include PhyloP Vertebrata (phyloP_V), SIFT, PROVEAN, SiPhy, GERP++, phastCons Vertebrata (phastCons_V), PhyloP Mammalia (phyloP_M) and phastCons Mammalia (phastCons_M), EVmutation (EVM) and LRT. The first column (ALL) provides the AUCs for all benign and deleterious variants (number provided in the blue top section of the table) that are scored by all methods compared (excluding LRT and EVM). In columns LRT and EVM, we intersect the ALL set with the set of variants that are scored by LRT and EVmutation, respectively. The IDR column contains the AUC for the subset of variants from ALL that are identified by ESpritz or IUpred to be located in intrinsically disordered protein regions. The highest AUCs achieved are highlighted in bold.

**Benign count**	**11,393**	**9,750**	**2,997**	**4,334**
**Deleterious count**	10,338	9,945	5,260	2,511
	**ALL**	**LRT**	**EVM**	**IDR**
**LIST**	**0.892**	**0.886**	**0.913**	**0.854**
**SIFT**	0.843	0.844	0.872	0.803
**PROVEAN**	0.838	0.841	0.848	0.808
**phyloP_V**	0.828	0.818	0.864	0.738
**SiPhy**	0.799	0.788	0.831	0.709
**phastCons_V**	0.787	0.772	0.815	0.709
**GERP++**	0.781	0.766	0.807	0.709
**phyloP_M**	0.772	0.757	0.796	0.729
**phastCons_M**	0.769	0.753	0.787	0.728
**EVmutation**			0.875	
**LRT**		0.804		

(b) AUC values for LIST and other methods that rely not only on conservation measures. These methods include Eigen, CADD, Fathmm-MKL, DANN, MutationTaster, Polyphen-2, MutationAssessor, GenoCanyon, fitCons HUVEC, fitCons H1-hESC, fitCons integrated (fitCons) and fitCons GM12878.

**Benign count**	**11,393**	**4,334**
**Deleterious count**	10,338	2,511
	**ALL**	**IDR**
**LIST**	**0.892**	**0.854**
**Eigen**	0.880	0.817
**fathmm-MKL**	0.855	0.784
**CADD**	0.854	0.777
**MutationTaster**	0.849	0.761
**PolyPhen-2**	0.842	0.803
**MutationAssessor**	0.837	0.804
**DANN**	0.816	0.758
**GenoCanyon**	0.733	0.667
**fitCons_HUVEC**	0.619	0.595
**fitCons**	0.618	0.620
**fitCons_H1-hESC**	0.614	0.598
**fitCons_GM**	0.600	0.606

Table 7: Contrasting AUC values using the HumVar test set

(a) AUC values for LIST and other methods that rely solely on conservation measures. These methods include PhyloP Vertebrata (phyloP_V), SIFT, PROVEAN, SiPhy, GERP++, phastCons Vertebrata (phastCons_V), PhyloP Mammalia (phyloP_M) and phastCons Mammalia (phastCons_M), EVmutation (EVM) and LRT. The first column (ALL) provides the AUCs for all benign and deleterious variants (number provided in the blue top section of the table) that are scored by all methods compared (excluding LRT and EVM). In columns LRT and EVM, we intersect the ALL set with the set of variants that are scored by LRT and EVmutation, respectively. The IDR column contains the AUC for the subset of variants from ALL that are identified by ESpritz or IUpred to be located in intrinsically disordered protein regions. The highest AUCs achieved are highlighted in bold.

**Benign count**	**8,397**	**7,532**	**2,517**	**3,113**
**Deleterious count**	9,054	8,547	5,613	1,407
	**ALL**	**LRT**	**EVM**	**IDR**
**LIST**	**0.900**	**0.899**	0.885	**0.898**
**SIFT**	0.883	0.883	0.888	0.871
**PROVEAN**	0.882	0.885	0.864	0.885
**phyloP_V**	0.857	0.857	0.843	0.857
**SiPhy**	0.825	0.822	0.806	0.826
**phastCons_V**	0.797	0.792	0.777	0.805
**GERP++**	0.788	0.784	0.770	0.781
**phyloP_M**	0.765	0.755	0.753	0.750
**phastCons_M**	0.751	0.744	0.726	0.764
**EVmutation**			**0.890**	
**LRT**		0.837		

(b) AUC values for LIST and other methods that rely not only on conservation measures. These methods include Eigen, CADD, Fathmm-MKL, DANN, MutationTaster, Polyphen-2, MutationAssessor, GenoCanyon, fitCons HUVEC, fitCons H1-hESC, fitCons integrated (fitCons) and fitCons GM12878.

**Benign count**	**8,397**	**3,113**
**Deleterious count**	9,054	1,407
	**ALL**	**IDR**
**LIST**	0.900	**0.898**
**Eigen**	**0.906**	0.894
**CADD**	0.887	0.866
**MutationAssessor**	0.882	0.878
**PolyPhen-2**	0.872	0.858
**fathmm-MKL**	0.870	0.872
**MutationTaster**	0.864	0.867
**DANN**	0.843	0.829
**GenoCanyon**	0.746	0.749
**fitCons_HUVEC**	0.539	0.447
**fitCons_H1-hESC**	0.533	0.522
**fitCons**	0.522	0.498
**fitCons_GM**	0.494	0.444

Table 8: Contrasting AUC values using the Cancer set.

(a) AUC values for LIST and other methods that rely solely on conservation measures. These methods include PhyloP Vertebrata (phyloP_V), SIFT, PROVEAN, SiPhy, GERP++, phastCons Vertebrata (phastCons_V), PhyloP Mammalia (phyloP_M) and phastCons Mammalia (phastCons_M), EVmutation (EVM) and LRT. The first column (ALL) provides the AUCs for all benign and deleterious variants (number provided in the blue top section of the table) that are scored by all methods compared (excluding LRT and EVM). In columns LRT and EVM, we intersect the ALL set with the set of variants that are scored by LRT and EVmutation, respectively. The IDR column contains the AUC for the subset of variants from ALL that are identified by ESpritz or IUpred to be located in intrinsically disordered protein regions. The highest AUCs achieved are highlighted in bold.

**Benign count**	**11,393**	**9,750**	**2,997**	**4,334**
**Deleterious count**	1,724	1,721	524	840
	**ALL**	**LRT**	**EVM**	**IDR**
**LIST**	**0.834**	**0.822**	**0.899**	**0.817**
**SIFT**	0.783	0.785	0.832	0.759
**PROVEAN**	0.774	0.777	0.810	0.774
**phyloP_M**	0.714	0.699	0.801	0.653
**phastCons_M**	0.711	0.691	0.858	0.618
**phyloP_V**	0.678	0.661	0.836	0.555
**GERP++**	0.675	0.656	0.812	0.569
**phastCons_V**	0.660	0.641	0.818	0.528
**SiPhy**	0.647	0.630	0.805	0.524
**EVmutation**			0.819	
**LRT**		0.655		

(b) AUC values for LIST and other methods that rely not only on conservation measures. These methods include Eigen, CADD, Fathmm-MKL, DANN, MutationTaster, Polyphen-2, MutationAssessor, GenoCanyon, fitCons HUVEC, fitCons H1-hESC, fitCons integrated (fitCons) and fitCons GM12878.

**Benign count**	**11,393**	**4,334**
**Deleterious count**	1,724	840
	**ALL**	**IDR**
**LIST**	**0.834**	**0.817**
**fitCons_GM**	0.823	0.809
**fitCons**	0.786	0.742
**PolyPhen-2**	0.780	0.767
**Eigen**	0.772	0.707
**MutationAssessor**	0.763	0.776
**fitCons_HUVEC**	0.763	0.699
**fitCons_H1-hESC**	0.746	0.692
**CADD**	0.738	0.659
**fathmm-MKL**	0.738	0.632
**MutationTaster**	0.723	0.586
**DANN**	0.716	0.670
**GenoCanyon**	0.606	0.538

Table 9: Median prediction scores and prediction performance before rescaling as a function of alignment depths.

AUC values for the predictions of all variants in optimization set 2 scored by LIST, PVM, PM1 and PM2 (before rescaling) as well as SIFT, and PROVEAN (PROV). Variants were placed in bins according to the alignment depths of the sequences in which they occur (outer left column). The counts of benign and deleterious variants, the percentage of deleterious variants, median scores for PVM, PM1, and PM2 for each bin as well as AUCs for PVM, PM1, PM2 and LIST before rescaling are provided in different columns. The last column provides the AUCs for predictions of variants in each bin provided by SIFT and PROVEAN. Bold numbers highlight the highest AUC achieved for each bin.

**Alignment Depth**		**Counts**		**Del.%**	**Median Scores before rescaling**			**AUC before rescaling**				**AUC**	
From	To	Benign	Del.		PVM	PM1	PM2	PVM	PM1	PM2	LIST	SIFT	PROV.
0	49	7,136	191	2.61%	0.32	0.88	0.33	0.667	0.622	0.666	**0.712**	0.646	0.631
50	99	4,070	269	6.20%	0.32	0.86	0.21	0.757	0.719	0.759	**0.813**	0.741	0.763
100	149	1,905	311	14.03%	0.45	0.87	0.30	0.754	0.75	0.779	**0.814**	0.742	0.745
150	199	922	181	16.41%	0.45	0.85	0.24	0.778	0.766	0.789	**0.844**	0.788	0.788
200	299	946	202	17.60%	0.45	0.83	0.04	0.793	0.777	0.775	**0.848**	0.79	0.773
300	399	571	150	20.80%	0.45	0.83	-0.10	0.802	0.81	0.794	**0.876**	0.833	0.789
400	∞	2,559	842	24.76%	0.32	0.77	-0.70	0.804	0.836	0.821	**0.874**	0.798	0.772
0	∞	18,109	2,146	10.59%				0.732	0.706	0.737	0.795	0.792	**0.807**

Table 10: Prediction performance after compensating for alignment depths.

AUC values for the predictions of variants in optimization set 2 by LIST and its three modules PVM, PM1 and PM2 (after rescaling) as well as SIFT and PROVEAN (PROV). Variants were placed in bins according to the alignment depths. Bold numbers highlight the highest AUC achieved.

Alignment Depth		AUC after rescaling				SIFT	PROV
From	To	PVM	PM1	PM2	LIST		
0	49	0.710	0.663	0.72	**0.756**	0.646	0.631
50	99	0.774	0.724	0.766	**0.814**	0.741	0.763
100	149	0.763	0.747	0.779	**0.816**	0.742	0.745
150	199	0.775	0.765	0.775	**0.842**	0.788	0.788
200	299	0.803	0.779	0.773	**0.854**	0.79	0.773
300	399	0.814	0.808	0.801	**0.876**	0.833	0.789
400	∞	0.810	0.841	0.826	**0.877**	0.798	0.772
0	∞	0.840	0.832	0.823	**0.880**	0.792	0.807

Supplementary Note 1

Comparison with other methods that predict the deleteriousness of human variants

We compared LIST with two broad categories of methods: (i) methods that rely solely on measures derived from multiple sequences alignment (MSA) and (ii) methods that, in addition to MSA derived measures, consider features derived from functional genomics data, available gene annotations and/or orthogonal prediction methods. Methods in category (i) can be subdivided further into methods that rely mainly on variant frequency and methods that exploit phylogenetic relationships among preselected subsets of species in order to determine departures from neutral substitution rates. It is important to note that orthogonal methods for the identification of deleterious human variants exist that rely on allele frequency in the human population[1-3]. Although successful, the scores of these methods are highly influenced by allele frequency and not of direct value for the evaluation of the new conservation measures introduced here.

We contrasted LIST prediction performance against the leading predictors in each of the two main categories. For category (i), we used SIFT[4], PROVEAN[5] and EVmutation[6] as representative methods that exploit variant frequencies as well as phyloP[7], SiPhy[8], GERP++ [9], phastCons[10] and LRT[11], which exploit phylogenetic relationships. For category (ii), we used Eigen[12], CADD[13], Fathmm-MKL[14], DANN[15], MutationTaster[16], Polyphen-2[17], MutationAssessor[18], GenoCanyon[19], and fitCons[20]. Both PhyloP and phastCons provide two different scores, the "_V" suffix implies the score is computed based on phylogenetic trees rooted at Vertebrata, and "_M" is for those based on trees rooted at Mammalia. FitCons provides four scores. Three scores are based on three different cell types: fitCons_HUVEC (human umbilical vein epithelial cells), fitCons_H1-hESC (H1 human embryonic stem cells), and fitCons_GM (lymphoblastoid cells, GM12878). The forth score, fitCons, is an integrated score of the three cell type scores. Many of these methods have recently been used in a benchmark study that compared deleteriousness predictions for coding and non-coding human variants[21]. Scores for the different predictors were downloaded from dbNSFPv3.5[22], except for EVmutation scores, for which we downloaded scores from its website.

When contrasting the performance of these predictors, we only used those variants that map to SwissProt proteins that have less than 50% identity to the proteins used in the optimization of LIST. In contrast, variants used in the optimization of these predictors are not excluded (as they are not always known), which results in a disadvantage for LIST in the comparison.

Supplementary Note 2

Assessing the influence of the frequency cut-off used in the definition of benign variants

It has been reported recently that common/benign variants with high allele frequency (e.g. AF > 50%) can be separated from deleterious ones with higher contrast compared to variants with modest allele frequency[6] (e.g. AF > 10%). Therefore, we compared the predictions of LIST with other methods using different allele frequency cut-offs in the definition of the benign class for the ClinVar/ExAC test set (Supplementary Table 4). Consistent with the prediction results reported for EVmutation[6], the contrast in LIST scores between deleterious and benign variants increases with the allele frequency cut-off (Supplementary Figure 3). Thus, the separation power provided by LIST is bigger at higher AF cut-off values. More importantly, LIST outperforms all contested predictors (Supplementary Table 4). At an AF cut-off >= 50%, for instance, LIST reaches an AUC of 0.971, which is higher than that of the leading category (i) method SIFT (0.930), and the leading category (ii) method Eigen (0.947). However, it is a common practice[23] to use allele frequencies smaller than 1% to identify the variants that are under purifying selection and consider those with higher frequency as neutral polymorphisms, i.e. benign. To be consistent with that practice, all of the following results are generated using test sets in which an AF >= 1% was used to define a putative benign class.

Supplementary Note 3

Assessing the influence of the optimization process on scoring

Most predictors tend to have a superior performance in predicting variants used in their optimization when compared to novel variants, i.e. deleterious (benign) variants used during optimization tend to be scored higher (lower) compared to previously unseen ones. This difference in scoring can impede the identification of novel variants.

When we designed the LIST algorithm, we took steps to limit the inflation of its optimization data. Mainly, we used a hierarchical learning approach to learn features separately, enabling us to rely on simple learning tools featuring low VC-dimension[24] (e.g. linear classifiers) that pose limited risk of over-scoring variants used in the optimization. In addition, we used deleterious training variants that were identified based on allele frequency instead of annotation (optimization set 1), which also provided us with larger numbers of positive training variants.

As a control for the influence of the optimization data on scoring, we first tested how much the prediction of the deleteriousness of variants change when variants used for testing are also used for optimization (training). Thus, we compared the reported performance of LIST on variants mapped to proteins in set B (Supplementary table 5 a first row) with the one LIST achieves when variants mapped to proteins in set B are also used in the optimization process (Supplementary table 5 a second row). We also tested how the performance changes when variants mapped to proteins in set A are used in testing (Supplementary table 5 a last two rows), i.e. the variants used for testing changed. We see that the AUC is virtually the same when using either variants mapped to proteins in set B or A for optimization. Overall, this analysis demonstrates that LIST is unlikely to score known variants with deleterious effect much higher than unseen ones, and that its performance does not depend on the selection of variants used for optimization and testing.

Supplementary Table 5 a also shows that variants in set B are slightly more separable than those in set A (AUCs are always slightly higher when variants in set B are tested), which is likely a result of clustering sequences at 50% identity and then dividing the clusters at random into two sets A and B. Importantly, most of the predictors that we compared LISTS with also perform better on variants in set B (Supplementary Table 5 b).

Supplementary Note 4

Compensating for alignment depth

We computed the range of PM2 scores at each alignment depth using the optimization set 2. For each alignment depth ε, we stored all PM2 scores with alignment depth in the range ε - 10 to ε + 10 in an array and computed the percentage of deleterious variants as ($pdel_{\varepsilon}$). Then, we sorted this array and used the score at 20% as lower boundary, L_boundary, and that at 80% as upper boundary, U_boundary.

When evaluating query sequences, for each position at alignment depth ε, we computed the un-correlated PM2 score $score_{UPM2}$ as:

$$\mathrm{score_{UPM2}} = (\mathrm{score_{PM2}} - \mathrm{L\_boundary}_{\varepsilon}) \Big/ (\mathrm{U\_boundary}_{\varepsilon} - \mathrm{L\_boundary}_{\varepsilon}) \quad (1)$$

Where $score_{PM2}$ is the PM2 score that is inversely correlated with alignment depth.

Then, we accounted for enrichment in deleterious variants, such that the *"probability like"* scores of module M_x at alignment depth ε, $p\_score_{Mx,\varepsilon}$ is:

$$\mathrm{p\_score_{Mx,\varepsilon}} = \mathrm{score_{Mx,\varepsilon}} * \mathrm{pdel}_{\varepsilon} \quad (2)$$

Where M_x is either PVM, PM1, or UPM2.

Supplementary Note 5

Redistributing scores to fit a target distribution

The idea is to alter the numerical values of a set of scores, SCORES, in the range [0 ... 1] from an unknown distribution to fit some desired target distributing without modifying their ranks within SCORES. In this work, we used this redistribution process three times; twice to redistribute the PM and CORE sets to fit a Normal distribution centered at the Bayes rule identity element (0.5) with a variance of 0.01, $N(\mu = 0.5, \sigma^2 = 0.01)$, and the third time to redistribute the final LIST score to fit a uniform distribution. This redistribution process involves two steps: first, we use a training data set (scores generated from optimization set 2) to learn a mapping function that map every value in the training set (unknown distribution) into a different value that reflect the target distribution without altering its rank, and then we use that mapping function for transforming any value from the input distribution to the target distribution. Supplementary figure 9 shows the mapping function learned for mapping the PM scores on optimization set 2 to $N(\mu = 0.5, \sigma^2 = 0.01)$, and supplementary figure 10 shows the probability density function of the benchmarking ClinVar/ExAC PM scores before and after redistribution.

Learning the mapping function: we created an array, CDF, of size 10,001 and filled it with the cumulative distribution of our target distribution. So, CDF[0]=0 and CDF[10000]=1. Then, we sorted SCORES in an ascending order. Consequently, the index of each value in SCORES is its rank. We created a third array MAPPING with a size equal to CDF, and we assigned MAPPING with the mapping values using CDF and SCORES as follows:

For each i in the range from zero to cdf_last:

$$\mathrm{X} = \mathrm{SCORES}[\mathrm{CDF}[\mathrm{i}] * \mathrm{scores\_last}] \tag{3}$$

$$\mathrm{MAPPING}[\mathrm{X} * \mathrm{cdf\_last}] = 0.025 + 0.95 * (\mathrm{i}/\mathrm{cdf\_last}) \tag{4}$$

Where:

scores_last is the size of the SCORES set minus one.

cdf_last is the size of the CDF set minus one.

Locations in MAPPING that were not assigned a value are then given a value based on their closest neighbors, linearly.

References

1 Dong, C. *et al.* Comparison and integration of deleteriousness prediction methods for nonsynonymous SNVs in whole exome sequencing studies. *Hum Mol Genet* **24**, 2125-2137, doi:10.1093/hmg/ddu733 (2015).

2 Jagadeesh, K. A. *et al.* M-CAP eliminates a majority of variants of uncertain significance in clinical exomes at high sensitivity. *Nat Genet* **48**, 1581-1586, doi:10.1038/ng.3703 (2016).

3 Rogers, M. F. *et al.* FATHMM-XF: accurate prediction of pathogenic point mutations via extended features. *Bioinformatics* **34**, 511-513, doi:10.1093/bioinformatics/btx536 (2018).

4 Ng, P. C. & Henikoff, S. SIFT: Predicting amino acid changes that affect protein function. *Nucleic Acids Res* **31**, 3812-3814 (2003).

5 Choi, Y., Sims, G. E., Murphy, S., Miller, J. R. & Chan, A. P. Predicting the functional effect of amino acid substitutions and indels. *PLoS One* **7**, e46688, doi:10.1371/journal.pone.0046688 (2012).

6 Hopf, T. A. *et al.* Mutation effects predicted from sequence co-variation. *Nat Biotechnol* **35**, 128-135, doi:10.1038/nbt.3769 (2017).

7 Hubisz, M. J., Pollard, K. S. & Siepel, A. PHAST and RPHAST: phylogenetic analysis with space/time models. *Brief Bioinform* **12**, 41-51, doi:10.1093/bib/bbq072 (2011).

8 Garber, M. *et al.* Identifying novel constrained elements by exploiting biased substitution patterns. *Bioinformatics* **25**, i54-62, doi:10.1093/bioinformatics/btp190 (2009).

9 Davydov, E. V. *et al.* Identifying a high fraction of the human genome to be under selective constraint using GERP++. *PLoS Comput Biol* **6**, e1001025, doi:10.1371/journal.pcbi.1001025 (2010).

10 Siepel, A. *et al.* Evolutionarily conserved elements in vertebrate, insect, worm, and yeast genomes. *Genome Res* **15**, 1034-1050, doi:10.1101/gr.3715005 (2005).

11 Chun, S. & Fay, J. C. Identification of deleterious mutations within three human genomes. *Genome Res* **19**, 1553-1561, doi:10.1101/gr.092619.109 (2009).

12 Ionita-Laza, I., McCallum, K., Xu, B. & Buxbaum, J. D. A spectral approach integrating functional genomic annotations for coding and noncoding variants. *Nat Genet* **48**, 214-220, doi:10.1038/ng.3477 (2016).

13 Kircher, M. *et al.* A general framework for estimating the relative pathogenicity of human genetic variants. *Nat Genet* **46**, 310-315, doi:10.1038/ng.2892 (2014).

14 Shihab, H. A. *et al.* An integrative approach to predicting the functional effects of non-coding and coding sequence variation. *Bioinformatics* **31**, 1536-1543, doi:10.1093/bioinformatics/btv009 (2015).

15 Quang, D., Chen, Y. & Xie, X. DANN: a deep learning approach for annotating the pathogenicity of genetic variants. *Bioinformatics* **31**, 761-763, doi:10.1093/bioinformatics/btu703 (2015).

16 Schwarz, J. M., Rodelsperger, C., Schuelke, M. & Seelow, D. MutationTaster evaluates disease-causing potential of sequence alterations. *Nature methods* **7**, 575-576, doi:10.1038/nmeth0810-575 (2010).

17 Adzhubei, I., Jordan, D. M. & Sunyaev, S. R. Predicting functional effect of human missense mutations using PolyPhen-2. *Current protocols in human genetics* **Chapter 7**, Unit7.20, doi:10.1002/0471142905.hg0720s76 (2013).

18 Reva, B., Antipin, Y. & Sander, C. Predicting the functional impact of protein mutations: application to cancer genomics. *Nucleic Acids Res* **39**, e118, doi:10.1093/nar/gkr407 (2011).

19 Lu, Q. *et al.* A statistical framework to predict functional non-coding regions in the human genome through integrated analysis of annotation data. *Scientific reports* **5**, 10576, doi:10.1038/srep10576 (2015).

20 Gulko, B., Hubisz, M. J., Gronau, I. & Siepel, A. A method for calculating probabilities of fitness consequences for point mutations across the human genome. *Nat Genet* **47**, 276-283, doi:10.1038/ng.3196 (2015).

21 Drubay, D., Gautheret, D. & Michiels, S. A benchmark study of scoring methods for non-coding mutations. *Bioinformatics* **34**, 1635-1641, doi:10.1093/bioinformatics/bty008 (2018).

22 Liu, X., Wu, C., Li, C. & Boerwinkle, E. dbNSFP v3.0: A One-Stop Database of Functional Predictions and Annotations for Human Nonsynonymous and Splice-Site SNVs. *Human mutation* **37**, 235-241, doi:10.1002/humu.22932 (2016).

23 MacArthur, D. G. *et al.* Guidelines for investigating causality of sequence variants in human disease. *Nature* **508**, 469-476, doi:10.1038/nature13127 (2014).

24 Vapnik, V. N. & Chervonenkis, A. Y. in *Measures of Complexity* (eds V. Vovk, H. Papadopoulos, & A. Gammerman) 11-30 (Springer International Publishing, 2015).
